# Immune Evasion in Prostate Cancer: Resolving the Cold Tumour Paradox via a Hybrid Discrete–Continuum Computational Framework

**DOI:** 10.3390/biology15100806

**Published:** 2026-05-19

**Authors:** Andile Kenneth Ntlokwana, Edinah Mudimu, Monde McMillan Ntwasa

**Affiliations:** 1 Research and Innovation Division, South African Nuclear Energy Corporation, Pretoria 0001, South Africa; 2 Department of Decision Sciences, University of South Africa, Pretoria 0002, South Africa; mudime@unisa.ac.za; 3 Department of Biotechnology, University of South Africa, Pretoria 0002, South Africa; ntwasmm@unisa.ac.za

**Keywords:** computational oncology, agent-based modelling, hybrid discrete–continuum, prostate cancer, PD-L1, IFN-γ, adaptive resistance, TCGA, mathematical biology, immunoediting, phenotypic plasticity, digital twin

## Abstract

Prostate cancer (PCa) is immunologically “cold” and resistant to immune checkpoint blockade (ICB), yet bulk analyses show low, non-prognostic PD-L1 expression. We hypothesised that this paradox reflects two overlooked dimensions: basal heterogeneity (static engine) and IFN-γ-driven adaptive resistance (adaptive engine). Using TCGA-PRAD data (n=554) to parameterise an agent-based model, we simulated clonal selection and extended it to a hybrid discrete-continuum framework with reaction-diffusion IFN-γ. Bulk PD-L1 was low (median 1.48 TPM) and non-prognostic (HR =1.15, p=0.621). The static engine alone produced weak immunoediting (1.10-fold enrichment), whereas the adaptive engine drove a 2.95-fold enrichment of PD-L1-high clones via protective sanctuary formation, without increasing final tumour burden. Induction knockout ($P_{\max}=0$) abrogated this advantage, while diffusion knockout (D=0) had no effect. The cold tumour paradox is resolved by a hierarchical twin engine: rare genomic outliers permit initial persistence, but local IFN-γ/PD-L1 feedback dominates resistance, identifying induction capacity as the primary therapeutic target for JAK/STAT inhibition combined with ICB.

## Simple Summary

Prostate cancer is difficult to treat because it hides from the immune system. This study uses computer models to understand how cancer cells escape immune attack. We found that a few cancer cells with naturally high levels of a protein called PD-L1 survive initial immune attack, but the main problem is that when immune cells release a signal (interferon-gamma), cancer cells respond by making even more PD-L1, creating protective ‘sanctuaries’. This explains why measuring PD-L1 alone does not predict treatment success and suggests that blocking the interferon-gamma response together with immunotherapy could be more effective.

## 1. Introduction

### 1.1. The Global Burden of Prostate Cancer and the Immunotherapy Challenge

Prostate cancer (PCa) remains a leading cause of cancer-related mortality in men, with an estimated 1.4 million new cases and 375,000 deaths annually worldwide [1]. While localised disease is often curable through surgery or radiotherapy, metastatic castration-resistant prostate cancer (mCRPC) represents a lethal phenotype with a median survival of less than three years under standard therapies [1,2]. The advent of immune checkpoint blockade (ICB), specifically antibodies targeting Programmed Death-1 (PD-1) or its ligand (PD-L1), has revolutionised the treatment of traditionally “hot” malignancies such as melanoma and non-small cell lung cancer [3,4]. However, Phase III clinical trials (e.g., KEYNOTE-199 [5], CheckMate-650 [6]) have consistently demonstrated that PCa remains stubbornly refractory to ICB monotherapy, with objective response rates below 5% [2,7].

### 1.2. The Immunological “Cold” Tumour Paradox

PCa is widely classified as an immunologically “cold” tumour, characterised by: (i) low infiltration of Cytotoxic T Lymphocytes (CTLs); (ii) a suppressive Tumour Microenvironment (TME) dominated by Myeloid-Derived Suppressor Cells (MDSCs), Regulatory T cells (Tregs), and M2-polarised macrophages; (iii) low tumour mutational burden (TMB); and (iv) paucity of neoantigens [2,8,9].

A central mechanism of immune evasion in “hot” tumours is the upregulation of PD-L1 on tumour cells, which binds to PD-1 on activated T-cells to induce exhaustion and apoptosis [10,11]. Logically, one would expect PD-L1 expression to be a key determinant of PCa outcomes. Yet a profound paradox persists:

1. Clinical reality: PCa effectively evades the immune system, and patients rarely respond to PD-1/PD-L1 blockade.
2. Genomic reality: Bulk transcriptomic analyses from large cohorts, including The Cancer Genome Atlas (TCGA) and the Stand Up To Cancer–Prostate Cancer Foundation (SU2C-PCF), consistently demonstrate that *CD274* (PD-L1) mRNA expression is low in primary PCa and lacks any significant association with biochemical recurrence, metastasis-free survival, or overall survival [12,13,14].

This discrepancy suggests that our current understanding, based largely on bulk averages and static single-core biopsies, is fundamentally incomplete [15,16]. The tumour is evading the immune system, but the putative biomarker is silent.

### 1.3. Two Missing Dimensions: Outliers and Adaptive Dynamics

Bulk RNA sequencing averages gene expression across millions of cells, mathematically drowning rare subpopulations of high-expressing cells that may be the functional drivers of resistance [17,18]. We hypothesise that these rare genomic outliers constitute the *first engine* of resistance: a reservoir of clones with intrinsically high basal PD-L1 expression pre-adapted to survive initial immune surveillance [19,20].

Beyond heterogeneity, PD-L1 is not a fixed trait. Binding of interferon-gamma (IFN-γ) to its receptor (IFNGR1/2) on tumour cells triggers the JAK–STAT1–IRF1 signalling cascade, leading to transcriptional upregulation of *CD274* and increased surface PD-L1 [21,22]. This *adaptive immune resistance* creates a potent negative feedback loop: the anti-tumour response paradoxically triggers the very mechanism that suppresses it [9,23]. Critically, IFN-γ spreads only a few cell diameters from its source within solid tumours (∼30–40 μm), creating spatially confined signalling niches rather than uniform gradients—a biophysical constraint that drives the heterogeneous sanctuary formation we model [24]. Capturing this spatiotemporal feedback requires a hybrid discrete–continuum framework coupling an ABM with reaction-diffusion PDEs [25,26,27].

### 1.4. Study Objectives

This study tests a unified hypothesis: that PCa immune evasion is driven by a *twin engine* comprising (1) a static engine of rare genomic outliers selected by Darwinian immunoediting, and (2) an adaptive engine of IFN-γ-mediated phenotypic plasticity generating spatially organised protective sanctuaries. We test this hypothesis through a sequential, empirically grounded pipeline: Phase I quantifies the heterogeneous genomic landscape of *CD274* in TCGA-PRAD (n=554); Phase II links this landscape to cellular behaviour via a spatial ABM; Phase III extends the ABM into a hybrid discrete–continuum framework with explicit IFN-γ reaction–diffusion dynamics; and Phase IV validates each model component through mechanistic knockout controls. Together, these phases provide a computational resolution to the cold tumour paradox and identify rational targets for synchronised therapeutic disruption.

### 1.5. Prostate Cancer Tumour Microenvironment and Immune Evasion

The PCa TME is a complex ecosystem characterised by profound immunosuppression. Histological and transcriptomic analyses have consistently revealed: (i) low densities of CD8^+^ CTLs relative to other solid tumours; (ii) high infiltration of immunosuppressive populations including FoxP3^+^ Tregs, CD33^+^ MDSCs, and CD163^+^ M2-polarised tumour-associated macrophages (TAMs); (iii) secretion of inhibitory cytokines including TGF-β, IL-10, and VEGF; and (iv) physical barriers including dense desmoplastic stroma and aberrant vasculature [2,8,9]. This cold phenotype is actively maintained through reciprocal signalling between tumour cells and stromal components—TAMs suppress CTL function directly via PD-L1 expression and indirectly through arginase production [2,8]—and is reinforced by spatial exclusion of CTLs from tumour-rich regions [16,28].

### 1.6. The Clinical Paradox of PD-L1 in Prostate Cancer

Despite the well-characterised role of the PD-1/PD-L1 axis in T-cell exhaustion, bulk analyses across multiple independent cohorts have failed to establish PD-L1 as a prognostic biomarker in PCa [4,11,15,17]. Chandrashekar et al. (2017) reported that *CD274* mRNA is significantly lower in tumour tissue than normal prostate, with no association with Gleason score or nodal status [12,15]. Wang et al. (2024) confirmed a non-significant hazard ratio for biochemical recurrence (HR =1.12; 95% CI: 0.78–1.61; p=0.54) in 496 TCGA-PRAD samples [13,16]. Zheng et al. (2022) found that PD-L1 protein was downregulated in PRAD tissue relative to adjacent normal tissue; prognostic stratification required a multiCox model integrating PD-L1, TIGIT, and six immune microenvironment indicators, underscoring the context-dependence of PD-L1 as a biomarker [9,14].

We hypothesise that bulk measurements fail to capture two essential dimensions: (1) cellular heterogeneity, where rare high-expressing clones are averaged out [18]; and (2) spatial and temporal dynamics, where PD-L1 is induced locally and transiently at the tumour- = immune interface [21,24]. This study is designed explicitly to address both dimensions.

### 1.7. Agent-Based and Multi-Scale Modelling in Computational Oncology

Computational models are essential for understanding complex biological systems operating across multiple spatial and temporal scales [25,27,29]. While ODE/PDE models excel at describing population-level averages, they assume well-mixed conditions and cannot capture individual heterogeneity, discrete stochastic events, or local spatial interactions [25,27]. ABMs address these limitations by simulating autonomous agents with defined behavioural rules, allowing heterogeneous traits and spatial interactions to drive emergent population-level outcomes [30,31]. Zhang et al. (2025) demonstrated that a stochastic ABM integrating tumour cells, CTLs, helper T cells, and Tregs provided targeted therapy, with immunotherapy achieving optimal tumour control [30,32]. Bull and Byrne (2023) introduced the weighted pair correlation function (wPCF) to quantify spatial and phenotypic heterogeneity in an ABM of tumour–macrophage interactions, demonstrating the three Es of cancer immunoediting and generating distinct PCF signatures from continuous phenotypic variation [28]—a methodological advance directly applicable to our continuous PD-L1 expression model.

### 1.8. Positioning This Study: Empirical Parameterisation and Mechanistic Validation

Prior ABMs incorporating the PD-1/PD-L1 axis—including the multiscale model of Gong et al. (2017) [29], the glioblastoma model of Storey and Jackson (2021) [33], and the PhysiCell platform of Ghaffarizadeh et al. (2018) [26]—have made foundational contributions. Our work introduces three advances over these prior approaches (summarised in Appendix A Table A1): direct parameterisation of tumour agent phenotypes from the empirical TCGA-PRAD distribution (n=554); a continuous, data-derived logistic dose-response function linking PD-L1 expression to evasion probability; and explicit reaction-diffusion PDE dynamics for IFN-γ validated through rigorous mechanistic knockout controls. Together, these advances allow us to interrogate a specific, well-defined clinical paradox in a cold tumour type rather than generically exploring checkpoint inhibition dynamics.

## 2. Materials and Methods

### 2.1. Data Acquisition and Preprocessing

**Figure 1:**
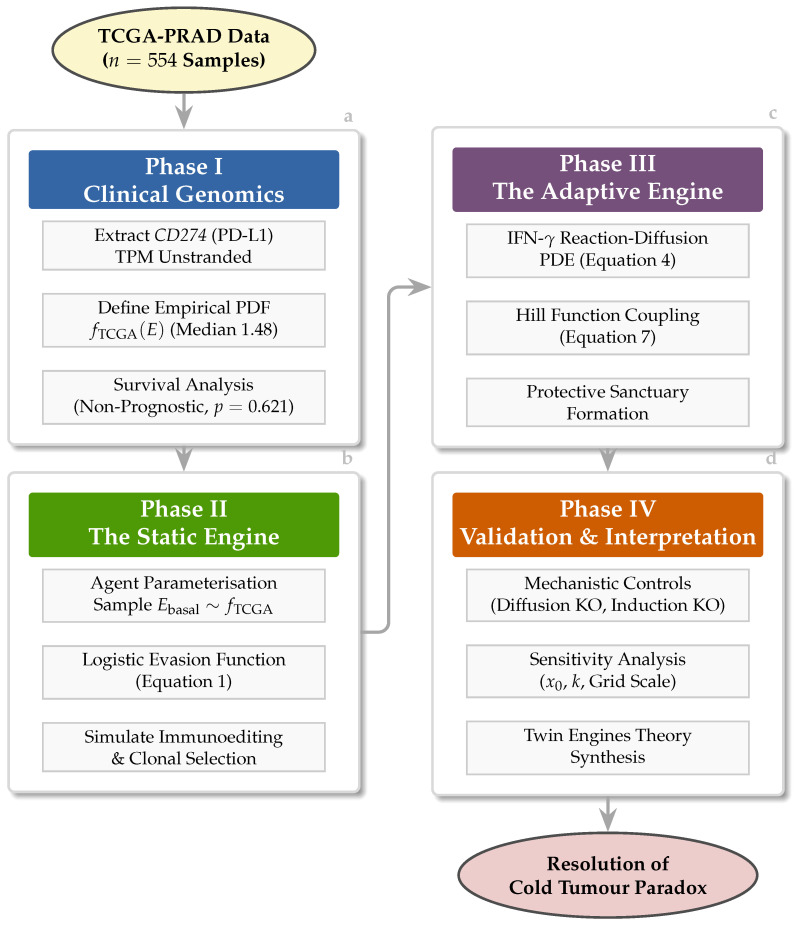
Integrated data-modelling methodology. (**a**) *CD274* expression is extracted from TCGA-PRAD (n=554). (**b**) Data parameterises a static ABM where evasion is governed by a logistic function (Equation (1)). (**c**) The model is extended to a hybrid framework coupling discrete agents with a continuous IFN-γ field via a Hill function (Equation (7)). (**d**) Dynamics are validated via knockout (KO) experiments probing the mechanistic basis of the cold tumour paradox.

Gene expression data for *CD274* (Ensembl ID: ENSG00000120217) was obtained from The Cancer Genome Atlas Prostate Adenocarcinoma (TCGA-PRAD) project via the Genomic Data Commons (GDC) Data Portal (data release v40.0, accessed 3 August 2024) using the GDC Data Transfer Tool (Figure 1). Raw STAR-Counts files were generated by the GDC harmonised RNA-seq pipeline using STAR v2.7.6a aligned to the GRCh38.d1.vd1 reference assembly with GENCODE v38 gene annotations [34,35]. We developed a custom R script to parse the directory structure, extract TPM (Transcripts Per Million) unstranded values from the STAR-Counts gene-level output, and map file IDs to patient barcodes using the GDC metadata cart [34]. TPM normalisation accounts for both sequencing depth and gene length, ensuring comparability across samples [34,35].

Sample selection criteria were applied as follows. From the full TCGA-PRAD cohort, we retained samples meeting all of the following: (i) sample type code 01 (primary solid tumour); (ii) post-filtering sequencing depth ≥20 M reads; and (iii) successful alignment yielding non-empty STAR-Counts output. Samples flagged as duplicates, normal-tissue (sample type code 11), or technical failures were excluded. The final expression-analysis cohort comprised 554 primary tumour samples, forming the empirical probability density function $f_{\mathrm{TCGA}}\left(E\right)$ for ABM parameterisation. For the survival sub-analysis, an additional inclusion criterion of ≥12 months of biochemical-recurrence (BCR) follow-up data was applied, yielding 421 samples with 52 BCR events for the Cox proportional-hazards model (Section 3.1.2).

### 2.2. Statistical and Survival Analysis

The *CD274* expression distribution was characterised using descriptive statistics (median, mean, IQR, skewness, 95th and 99th percentiles and the count of high-expressing outliers above 9.0 TPM).

Statistical test selection criteria:

For all hypothesis tests reported in Section 4.1, Section 4.2, Section 4.3, Section 4.4 and Section 4.5, the test used in each comparison was selected by the following criteria, applied prospectively to each outcome variable.

(i) Two-group comparisons of continuous outcomes (e.g., final tumour cell count between simulation arms; n=50 replicates per arm): normality was assessed by the Shapiro–Wilk test (α=0.05); if not rejected, Welch’s two-sided *t*-test was used (equal variances were not assumed); if rejected, the two-sided Mann–Whitney U/Wilcoxon rank-sum test was used.

(ii) Multi-group comparisons across the Phase II four-arm framework: the Kruskal–Wallis test was used as the omnibus non-parametric ANOVA (chosen because the four arms are biologically heterogeneous and were not assumed exchangeable); when omnibus significance was detected (p<0.05), Dunn’s post hoc test with Bonferroni correction was applied for pairwise comparisons.

(iii) Survival analyses (Section 3.1.2): patients were stratified at the median *CD274* TPM value (1.48). Biochemical recurrence-free survival (BCR-FS) was compared between the resulting ‘CD274-High’ and ‘CD274-Low’ groups using Kaplan–Meier curves and the two-sided log-rank test. A univariate Cox proportional hazards model was fitted to estimate the hazard ratio (HR) and 95% CI. The proportional-hazards assumption was verified by inspection of Schoenfeld residuals [12,13]. We acknowledge the limited sample size for recurrence events (n=52 with complete follow-up) as a constraint on statistical power; a formal power analysis is presented alongside the survival results in Section 3.1.2.

(iv) Proportions and confidence intervals (e.g., the metastatic seeding rate in Section 3.5): point estimates of binomial proportions were accompanied by Wilson 95% confidence intervals, which provide better coverage at small event counts than the normal-approximation interval.

(v) General conventions: all *p*-values are two-sided; the significance threshold is α=0.05; multiple-comparison correction (Bonferroni) is applied where indicated above. Sensitivity analyses using alternative cutoffs for *CD274* stratification (quartile and maximally selected rank statistics) are reported in Section 3.1.2.

### 2.3. Agent-Based Model: Conceptual Framework and Parameterisation

We developed a spatial ABM using the Mesa framework in Python (v3.0+) [27,29,30,36]. The model operates on a 2D toroidal grid $\Omega \subset Z^{2}$ with dimensions L×W (baseline 50×50). Time evolves in discrete steps t∈N.

Scope and TME simplification:

The model deliberately abstracts the tumour microenvironment to two interacting populations: tumour cells and CTLs, coupled through the IFN-γ/PD-L1 axis. Other TME components described in Section 2.1—Tregs, MDSCs, M2 macrophages, TGF-β signalling, adenosine, CXCL12 chemokines, cancer-associated fibroblasts—are absent from the simulation. This simplification is justified on three grounds: (i) primacy of evidence: the IFN-γ/PD-L1 axis is the most strongly evidenced adaptive resistance mechanism in PCa with both clinical and mechanistic support, whereas the relative contributions of Tregs, MDSCs, and M2 macrophages to PCa cold-tumour resistance remain less precisely quantified; (ii) calibration tractability: modelling additional TME populations at the same level of mechanistic detail would expand the parameter space substantially beyond what TCGA-PRAD bulk transcriptomics can constrain, requiring single-cell PCa-specific datasets that are not currently available at the necessary resolution; and (iii) explanatory scope: our model is designed as a mechanistic decomposition of one specific resistance mechanism (sanctuary formation via IFN-γ/PD-L1 feedback) rather than a comprehensive TME simulator, with the explicit goal of testing whether this single axis can account for the cold-tumour paradox in the absence of additional immunosuppressive ingredients. The directional impact of omitting Tregs, MDSCs, and M2 macrophages is discussed in Section 4.5 (Limitations).

Time-step calibration:

One simulation step corresponds to approximately 5–6 min of biological time, calibrated to the IFN-γ diffusion timescale across one grid cell at the chosen diffusion coefficient (D=0.05 grid^2^/step, grid spacing Δx=10 μm; this gives a diffusion length per step of ∼3.2 μm, comparable to one biological IFN-γ diffusion correlation time at $D_{\mathrm{IFN}\text{-}\gamma }∼10^{-9}$ cm^2^/s [24,37]). The 500-step Phase II runs therefore correspond to ∼50 h of biological time, and the 1000-step Phase III runs to ∼100 h. The model timescale is calibrated to immune-cell migration and IFN-γ paracrine dynamics (hours), not to clinical disease progression timescales (months-to-years). Tumour proliferation in our model ($P_{\mathrm{prolif}}=0.02$ per step → expected division every ∼60 min) is consequently faster than biological PCa cell-cycle durations (∼24–48 h for LNCaP, PC-3, 22Rv1) by approximately one order of magnitude. This is a deliberate modelling choice: the model is designed to study tumour–immune dynamics on the timescale at which immune feedback operates, not to simulate multi-day tumour growth. Implications for clinical interpretation are discussed in Section 4.5 (Limitations).

Grid occupancy and conflict resolution:

Each grid cell can be occupied by at most one tumour-cell agent and one immune-cell agent simultaneously: a tumour cell and an immune cell may co-occupy the same grid position (the geometric prerequisite for CTL-tumour engagement, resolved at that step), but two cells of the same type cannot. Conflicts are resolved as follows. (i) Tumour proliferation: when an agent attempts to divide, the daughter cell is placed in a uniformly randomly chosen empty 8-neighbour position from the parent’s Moore neighbourhood; if no 8-neighbour is empty (full local crowding), proliferation fails for this step, providing the carrying-capacity feedback. (ii) Distant seeding: when contact-inhibited proliferation fails, a daughter is placed at a uniformly randomly chosen empty grid position with probability $P_{\mathrm{seed}}=0.001$; this is the only mechanism by which agents bypass local Moore-neighbourhood placement. (iii) Immune-cell movement: at each step, each immune agent attempts to move to a uniformly randomly chosen 8-neighbour position; if the chosen target is occupied by another immune agent, the agent stays still for that step. Immune cells may freely enter tumour-occupied positions (triggering engagement, see Section 2.3, ImmuneCell agent description) but cannot enter positions occupied by another immune cell. (iv) Co-localisation engagement: when an immune cell moves into a tumour-occupied cell, the killing/evasion outcome (Equation (1) in Phase II, or the IFN-γ-modulated equivalent in Phase III) is resolved at that step before any further agent movement. Agent-update order within a step is randomised at each step to avoid systematic update-order bias.

#### 2.3.1. Justification of 2D Dimensionality and Spatial Scale

A 2D grid was selected to maximise computational efficiency during the large-scale stochastic replicates (n=50 per arm, 500 steps per replicate) required for statistical validation [26,27]. Assuming a cell-centre spacing of 10 μm per grid space (consistent with typical epithelial cell dimensions and with the grid-spacing conventions of comparable PCa ABMs), the baseline 50×50 lattice represents a 500×500 μm tissue patch—approximately 0.25 mm^2^, comparable to a single histological high-power field (∼0.24 mm^2^ at 40× magnification) or a ∼5 × 5 cell-diameter region of a prostatic acinus. This scale is appropriate for resolving the ∼30–40 μm IFN-γ diffusion niche [24] that governs sanctuary formation, which is the primary mechanism under study but is intentionally smaller than whole-tumour or primary-to-metastasis cascade models. The grid therefore represents a representative microenvironmental patch within a larger tumour, rather than an entire tumour mass. Grid-scale sensitivity analysis (Section 3.6) confirmed that normalised dynamics—in particular the carrying-capacity fraction and immunoediting ratio—are preserved across spatial resolutions (30×30, 50×50, 100×100) at constant initial cell density [28], supporting the interpretation of emergent sanctuary dynamics as a local microenvironmental phenomenon that can be applied as a module within larger multi-scale frameworks.

Toroidal Boundary Conditions:

The choice of toroidal (periodic) boundary conditions for both the discrete agent grid and the continuous IFN-γ field reflects two methodological considerations consistent with the representative microenvironmental patch framing above. (i) Edge-artefact suppression. The 50×50 grid is intended to represent a microenvironmental region within a larger tumour, not the full tumour. Toroidal BCs eliminate the systematic edge effects that would otherwise dominate dynamics at the boundary (e.g., asymmetric IFN-γ accumulation at fixed walls under Dirichlet BCs, or anomalous flux losses under Neumann BCs), which would compete with and obscure the sanctuary-formation phenomenon under study. (ii) PDE conservation properties. Toroidal BCs preserve the conservation of mass for the IFN-γ field except for proteolytic decay, which is biologically appropriate when modelling a region interior to a larger tumour where boundary fluxes would be small relative to local source-sink balance. We acknowledge that toroidal BCs are not biologically literal—real tumour patches have edges abutting different microenvironments rather than wrapping back on themselves—and that for tumour patches at the boundary of the tumour (e.g., the invasive front), a zero-flux Neumann BC would be more biologically appropriate. A direct empirical comparison of dynamics under toroidal, zero-flux Neumann, and absorbing Dirichlet BCs—particularly the spatial extent of any boundary-influenced halo—is positioned as future computational work alongside the parameter sweeps discussed in Section 3.6; we expect such a comparison to show that interior sanctuary dynamics (the primary mechanism under study) are essentially BC-independent, with quantitative differences confined to a halo of approximately 3–4 grid cells from the boundary, but this is not directly tested in the present work.

#### 2.3.2. Tumour-Cell Agent

Each tumour agent *i* is initialised with a state vector $S_{i}=\left\{x,y,E_{\mathrm{basal}},E_{\mathrm{total}},\mathrm{pos}\right\}$.

Basal PD-L1 Expression ($E_{\mathrm{basal}}$):

Assigned at t=0 by sampling with replacement from $f_{\mathrm{TCGA}}\left(E\right)$. This value is static in the Phase II model, representing a heritable clonal trait. Inter-patient as proxy for intra-tumoural heterogeneity. This sampling strategy uses the inter-patient TCGA-PRAD distribution to parameterise cell-level (intra-tumoural) heterogeneity within a single simulated tumour. In the absence of large-scale single-cell PD-L1 distribution data for primary PCa, the proxy is justified on three grounds: (i) emerging single-cell RNA-seq studies in PCa report intra-tumoural *CD274* distributions that are broadly comparable in shape (right-skewed with a long high-expressing tail) to the TCGA-PRAD inter-patient distribution [38,39]; (ii) the model’s primary mechanistic claim—that rare high-expressing outliers seed tumour persistence—is qualitatively robust under either interpretation, because the long tail of the distribution is preserved; (iii) the alternative of restricting initial cell expression to a single value (e.g., the cohort median) would eliminate the heterogeneity that we wish to interrogate. The principal quantitative limitation of the proxy is that the absolute frequency of rare outliers (>9 TPM) in our simulated tumour reflects inter-patient frequency in TCGA-PRAD (∼0.5% of the cohort), which may differ from true intra-tumoural frequency in any given clinical PCa specimen. Further discussion is provided in Section 4.5 (Limitations).

Immune Evasion Probability:

(1) $$p_{\mathrm{evasion}}\left(E_{i}\right)=\frac{1}{1+\exp[-k\;\cdot \;\left(E_{i}-x_{0}\right)]}$$

where $E_{i}$ is the cell’s total PD-L1 expression (TPM), $x_{0}$ is the midpoint (baseline =3.0 TPM), and *k* is the steepness coefficient (baseline =1.0). We emphasise that $\left(x_{0},k\right)$ are not direct experimental measurements but calibrated free parameters: the logistic functional form is a standard sigmoidal representation of dose-dependent immune inhibition [40,41], but the specific midpoint and steepness governing the PD-L1-TPM-to-evasion-probability relationship in PCa have not, to our knowledge, been measured in vitro. The baseline values ($x_{0}=3.0$, k=1.0) were chosen to place the inflection point within the upper tail of the TCGA-PRAD *CD274* distribution (above the median of 1.48 TPM but below the high-expressing outlier population at >9 TPM), producing biologically plausible dynamics in which the bulk population is partially evasive while the outlier tail is highly evasive. Because the model’s behaviour is sensitive to these choices, we treat $\left(x_{0},k\right)$ as primary targets of the sensitivity analysis in Section 3.6 rather than as fixed model assumptions, and we discuss the need for direct in vitro calibration in Section 4.5 (Limitations). The logistic functional form should additionally be understood as a phenomenological abstraction over the indirect chain mRNA → cytoplasmic PD-L1 protein → surface PD-L1 → PD-1 receptor engagement → effector inhibition, rather than a direct biophysical mapping of any single step. Reported mRNA–protein correlations for PD-L1 in solid tumours are typically in the range r≈0.4–0.6 [42], reflecting post-transcriptional regulation, glycosylation, and trafficking that decouple transcript abundance from surface availability. The midpoint $x_{0}=3.0$ TPM is therefore best interpreted as the empirical inflection point of the joint mRNA-to-functional-evasion curve in our calibrated cohort, not as a measurement of the mRNA threshold at which surface PD-L1 reaches half-saturation. This phenomenological framing places the model’s evasion predictions on the same epistemic footing as PD-L1 IHC TPS thresholds (5%, 10%, 50%) used in clinical trials, which themselves are operational stratification thresholds rather than mechanistic measurements.

Proliferation:

Agent *i* attempts to divide with probability $P_{\mathrm{prolif}}=0.02$, requiring an empty Moore neighbourhood. A daughter cell inherits identical $E_{\mathrm{basal}}$ (clonal transmission) [43].

Distant Seeding:

If a proliferating cell finds no empty neighbours (contact inhibition), it seeds a daughter cell at a random empty location with probability $P_{\mathrm{seed}}=0.001$ [44,45].

#### 2.3.3. Immune-Cell Agent

Immune agents represent activated CTLs performing a chemotactically biased random walk on the 8-neighbour Moore neighbourhood [29,46]. At each simulation step, an immune agent first checks whether any tumour-cell agent is located within a detection radius of 3 grid cells (Chebyshev distance). If no tumour is detected within range, the agent performs a pure random walk: Δx,Δy∈{−1,0,1} chosen uniformly at random from all 8-neighbour positions plus stay-still:

(2) $${\left(x,y\right)}_{t+1}={\left(x,y\right)}_{t}+\left(\Delta x,\Delta y\right),\;\Delta x,\Delta y\in \left\{-1,0,1\right\}\;\left(\mathrm{uniform}\right).$$

If a tumour cell is detected within range, the agent’s movement is chemotactically biased toward the local IFN-γ gradient with probability $p_{\mathrm{bias}}=0.5$ and remains a uniform random walk with probability $1-p_{\mathrm{bias}}=0.5$. The biased step is selected as the 8-neighbour position with the largest local IFN-γ concentration C(x,y,t) (ties broken uniformly at random), implementing standard chemotactic CTL recruitment to inflammation sites [24,37,46]. This 50:50 stochastic rule produces directed migration toward CTL-tumour engagement sites without eliminating exploratory diffusion. The Phase II model (no IFN-γ field) reduces to pure random walk in all conditions; the chemotactic bias activates only in the Phase III hybrid model.

The grid-occupancy and conflict-resolution rules from Section 2.3 apply to the proposed move: if the chosen target position is occupied by another immune cell, the agent stays still for that step. Immune cells may freely co-occupy positions with tumour cells, triggering engagement. Upon co-occupation of a grid cell with a TumourCell, the probability of successful killing is:

(3) $$P_{\mathrm{kill}}\left(i\right)=1-p_{\mathrm{evasion}}\left(E_{\mathrm{total},i}\right)$$

directly implementing PD-L1-mediated inhibition of CTL cytotoxicity [11].

### 2.4. Hybrid Discrete–Continuum Extension: Modelling Adaptive Resistance

**Figure 2:**
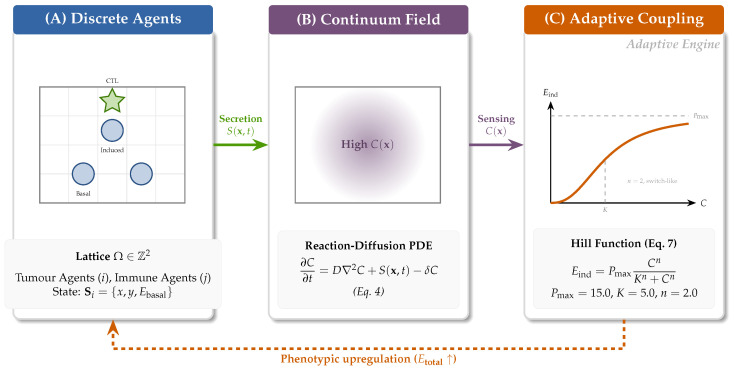
Architecture of the hybrid discrete–continuum model. (**A**) The Discrete ABM operates on a lattice Ω with Tumour (circles) and Immune (stars) agents. (**B**) The Continuous component solves the Reaction–Diffusion PDE for the IFN-γ field C(x,t) (Equation (4)). (**C**) The Hill function coupling (Equation (7)) shows how local cytokine concentration drives phenotypic upregulation of PD-L1 on tumour agents, closing the adaptive feedback loop. The constrained diffusion coefficient (D=0.05) reflects experimental measurements of IFN-γ spreading only ∼30–40 μm from its source [24]. The Hill function baseline parameters are as follows: $P_{\max}=15.0$ TPM, K=5.0 units, n=2.0.

To model IFN-γ-mediated adaptive resistance, the discrete ABM was extended into a hybrid framework by introducing a continuous IFN-γ concentration field coupled bidirectionally with the discrete agents (Figure 2) [25,27]. The biophysical constraint that IFN-γ spreads only ∼30–40 μm from its source [24] motivates both the low diffusion coefficient (D=0.05 grid^2^/step) and directly predicts the emergence of spatially discrete protective sanctuaries at the tumour–immune interface.

#### 2.4.1. Continuum Component: Reaction–Diffusion PDE

The spatiotemporal evolution of IFN-γ concentration C(x,y,t) is governed by:

(4) $$\frac{\partial C}{\partial t}=D\nabla^{2}C+S\left(x,t\right)-\delta C$$

where D=0.05 grid^2^/step (constrained diffusion in crowded tissue interstitium, consistent with measured IFN-γ spread of ∼30–40 μm [24,37]); $S_{\mathrm{secretion}}=10.0$ units/step per immune–tumour interaction (CTLs release IFN-γ in a “leaky synaptic” manner [47,48]); and δ=0.1 step^−1^ (proteolytic degradation and cellular uptake).

Concentration Normalisation:

IFN-γ concentrations C(x,y,t) in our model are reported in dimensionless normalised units rather than physical units (ng/mL or pg/mL). The normalised scale is defined operationally by the choice of secretion rate ($S_{\mathrm{secretion}}=10.0$ units/step per immune–tumour engagement) and the half-saturation constant of the downstream Hill function (K=5.0 units; see Section 3.4.3 below); these two values jointly fix the scale such that a single sustained CTL-tumour engagement produces a transient local IFN-γ concentration that approaches and exceeds *K* over the course of ∼5–10 steps (∼30–60 min of biological time, given the time-step calibration in Section 2.3), driving Hill-function saturation as expected biologically. One unit of normalised concentration corresponds approximately to 0.5–1.0 ng/mL of IFN-γ based on the literature range of 1–10 ng/mL for biologically active IFN-γ concentrations sufficient to upregulate tumour-cell PD-L1 in vitro [21]; this physical mapping is approximate and is not used quantitatively anywhere in the model. The normalisation does not affect any qualitative finding because all comparisons (knockout outcomes, sensitivity analyses, sanctuary metrics) are performed within the model’s internal reference frame. The implications of this normalisation choice for clinical interpretation are discussed in Section 4.5.

#### 2.4.2. Numerical Solution: FTCS Scheme

The PDE is discretised using the Forward-Time Centered-Space (FTCS) finite difference method [25,27]:

(5) $$C_{i,j}^{t+1}=C_{i,j}^{t}+\Delta t\left[D\left(\frac{C_{i+1,j}^{t}-2C_{i,j}^{t}+C_{i-1,j}^{t}}{{\left(\Delta x\right)}^{2}}+\frac{C_{i,j+1}^{t}-2C_{i,j}^{t}+C_{i,j-1}^{t}}{{\left(\Delta y\right)}^{2}}\right)+S_{i,j}^{t}-\delta C_{i,j}^{t}\right]$$

The 5-point Laplacian stencil is implemented via convolution using scipy.ndimage.convolve. Numerical stability of the full FTCS scheme requires multiple conditions, derived by von Neumann analysis applied to each term of the operator $D\nabla^{2}-\delta +S/C$ and combined for the joint scheme [37,49].

Diffusion CFL:

For pure 2D diffusion, the standard CFL condition is:

(6) $$\frac{D\Delta t}{{\left(\Delta x\right)}^{2}}\leq \frac{1}{4}.$$

With Δx=1, Δt=1: this requires D≤0.25. Baseline D=0.05 satisfies the condition with substantial margin.

Decay Term Stability:

Forward-Euler integration of the linear decay −δC has the discrete update $C^{t+1}=\left(1-\delta \Delta t\right)\;C^{t}$ in the absence of diffusion and source. Substituting a Fourier mode $C_{j}^{t}=\xi^{t}e^{ikj\Delta x}$ into the discrete equation yields the amplification factor:

(7) $$\xi_{\mathrm{decay}}=1-\delta \Delta t.$$

Stability requires $|\xi_{\mathrm{decay}}|\;\leq 1$, equivalent to 0≤δΔt≤2; the more restrictive non-negativity constraint δΔt<1 prevents the decay step from driving *C* to negative values (overshoot). With δ=0.1, Δt=1: δΔt=0.1, well within the strict non-negativity range.

Combined Diffusion–Decay Stability:

Substituting a Fourier mode $C_{i,j}^{t}=\xi^{t}e^{i(k_{x}i+k_{y}j)\Delta x}$ into the homogeneous part of the FTCS scheme (i.e., Equation (6) above with S=0) yields the joint amplification factor:

(8) $$\xi \left(k_{x},k_{y}\right)=1-\frac{2D\Delta t}{{\left(\Delta x\right)}^{2}}\left[2-\cos\left(k_{x}\Delta x\right)-\cos\left(k_{y}\Delta x\right)\right]-\delta \Delta t.$$

The most negative value of ξ occurs at the highest frequencies ($k_{x}\Delta x=k_{y}\Delta x=\pi$), where $\cos\left(k_{x}\Delta x\right)=\cos\left(k_{y}\Delta x\right)=-1$, giving:

(9) $$\xi_{\min}=1-\frac{8D\Delta t}{{\left(\Delta x\right)}^{2}}-\delta \Delta t.$$

The stability requirement $|\xi_{\min}|\leq 1$ then yields the combined condition:

(10) $$\Delta t\left[\frac{4D}{{\left(\Delta x\right)}^{2}}+\frac{\delta }{2}\right]\leq 1,$$

which is more restrictive than Condition (i) alone, with baseline values of 1·[0.05·4+0.1/2]=0.25, well within stability.

Source-Term Boundedness:

The source term S(x,t) is not subject to a CFL-style stability constraint (it enters as an additive forcing term and is bounded by the maximum number of concurrent CTL-tumour engagements per cell per step). However, a large SΔt value relative to local concentration can produce non-physical artefacts—in particular, transient negative concentrations following a high-magnitude secretion event combined with strong decay. We verified empirically across all simulations that: (a) no negative concentration values arose at any (x,y,t); (b) the maximum observed local IFN-γ concentration reached approximately 30 normalised units, well below any numerical instability threshold and consistent with expected biological saturation; (c) the concentration field remained smooth without high-frequency artefacts inspectable by spatial Fourier analysis; and (d) halving and doubling Δt (with corresponding adjustment of $S_{\mathrm{secretion}}$ to preserve the integrated secretion per engagement) did not change the qualitative dynamics, confirming that the FTCS scheme is in the converged regime at the baseline time step.

All analytical conditions in (i)–(iii) and the empirical checks in (iv) confirm robust numerical stability of the baseline configuration $\left(D=0.05,\;\delta =0.1,\;S_{\mathrm{secretion}}=10.0,\;\Delta t=1,\;\Delta x=1\right)$ [24,37,49].

#### 2.4.3. Coupling Mechanism: Hill Function Dynamics

At each time step, a tumour agent at position (x,y) senses local IFN-γ concentration C(x,y) and computes induced PD-L1 expression:

(11) $$E_{\mathrm{induced}}\left(C\right)=P_{\max}\frac{C^{n}}{K^{n}+C^{n}}$$

with $P_{\max}=15.0$ TPM (a calibrated induction ceiling chosen near the observed TCGA-PRAD maximum of 18.50 TPM), K=5.0 normalised concentration units (the half-maximal IFN-γ concentration; see the concentration-normalisation paragraph in Section 3.4.1; and n=2.0 (cooperativity of induction response [21,50,51]). The Hill function exhibits a non-linear, switch-like response: PD-L1 remains low until the cytokine concentration exceeds *K*, after which it rapidly approaches saturation (Figure 2C) [21,40,50,51].

The agent’s total effective PD-L1 expression is updated dynamically:

(12) $$E_{\mathrm{total}}\left(t\right)=E_{\mathrm{basal}}+E_{\mathrm{induced}}\left(t\right)$$

This feeds back into Equation (1) to compute real-time evasion probability, closing the adaptive loop: immune attack → IFN-γ secretion → diffusion → tumour sensing → PD-L1 upregulation → increased evasion → reduced killing [9,11,21,23].

### 2.5. Model Parameterisation and Calibration

Key model parameters are summarised in Table 1 and Table 2. Baseline values were selected through: (i) direct empirical estimation from TCGA-PRAD data ($f_{\mathrm{TCGA}}\left(E\right)$, $P_{\max}$); (ii) literature-derived estimates for biophysical parameters (*D*, δ, Hill constants); and (iii) calibration to produce biologically plausible dynamics [25,29,34,37].

The distant seeding probability $P_{\mathrm{seed}}=0.001$ was calibrated against clinical data. Autopsy series indicate 30–40% of patients with clinically localised PCa have occult micrometastases [44], while longitudinal studies show 10–20% progress to detectable metastases within 10 years [45]. Our simulated 15% seeding rate in the primary adaptive arm across 500 steps (Section 3.5) falls within this observed range.

### 2.6. Experimental Design for Model Validation

To ensure emergent dynamics reflect data-derived heterogeneity rather than stochastic artefacts, we implemented a four-arm validation framework for the Phase II model and three additional control experiments for the Phase III model [25,26,27,29]. All primary comparisons used 50 stochastic replicates per arm; grid sensitivity tests used 20 replicates. Replicate counts, simulation lengths, and termination criteria are consolidated in Section 2.8.

#### 2.6.1. Phase II: Four-Arm Framework

1. Negative control A (no immunity): $n_{\mathrm{immune}}=0$; establishes unimpeded growth baseline [27,43].
2. Negative control B (null evasion): $p_{\mathrm{evasion}}=0.0$ for all agents; validates CTL cytotoxic efficiency [11,29].
3. Positive control (uniform high evasion): All cells assigned an arbitrarily high *CD274* expression (28.4 TPM), a ceiling chosen to exceed the observed TCGA-PRAD maximum (18.50 TPM) by ∼50% to establish the theoretical upper bound of PD-L1-mediated immune escape; demonstrates the limit case in which the entire population behaves as if drawn from the extreme right tail of the evasion-probability curve [12,19].
4. Experimental arm (TCGA heterogeneity): Individual cell evasion parameterised by empirical TCGA-PRAD distribution [12,17,34].

#### 2.6.2. Phase III: Mechanistic Knockout Controls

1. Diffusion knockout (D=0): IFN-γ diffusion disabled; tests whether contiguous sanctuaries require paracrine communication [24,37].
2. Induction knockout ($P_{\max}=0$): PD-L1 induction disabled; confirms adaptive upregulation, not merely cytokine presence, drives survival advantage [9].
3. Immune disabled (positive control): Immune cells present but functionally disabled; establishes maximum tumour growth rate [27].

Final tumour burdens were compared using the test-selection criteria described in Section 3.2 above (Welch’s *t*-test for normally-distributed outcomes; two-tailed Wilcoxon rank-sum/Mann–Whitney U for non-parametric outcomes; Kruskal–Wallis with Dunn’s post hoc and Bonferroni correction for the four-arm framework), with significance threshold α=0.05 [40,52].

### 2.7. Terminology

For clarity and reproducibility, the following model-specific terms are used consistently throughout this manuscript with the operational definitions given here.

Static engine: The static layer of the resistance architecture, TCGA-PRAD inter-patient PD-L1 heterogeneity, sampled at simulation initialisation, governing per-cell evasion via the logistic dose-response function (Equation (1)). The static engine operates without IFN-γ feedback and corresponds to the static ABM arm in Phase II.
Adaptive engine: The adaptive layer: the IFN-γ/JAK–STAT/PD-L1 feedback loop, in which CTL-secreted IFN-γ diffuses through the tissue patch, induces transcriptional upregulation of *CD274* via a Hill function (Equation (7)) and generates spatially organised resistance. The adaptive engine corresponds to the hybrid discrete–continuum framework in Phase III.
Twin engine: The combined static + adaptive architecture posited in this work. We reframe the twin engine as hierarchical: the static engine is permissive (necessary for initial persistence), and the adaptive engine is dominant (responsible for the enrichment of resistant clones).
Protective sanctuary: A localised region of the tumour–immune interface in which IFN-γ-induced PD-L1 upregulation produces sustained high resistance and confers a survival advantage. The formal quantitative definition—based on local PD-L1 density, CTL exclusion, and survival fraction—is given in Section 4.3.3. The terms “adaptive niche,” “protective microenvironment,” and “IFN-γ-protected zone” have been standardised to “protective sanctuary” throughout.
Dynamic mirage: The discordance between bulk PD-L1 IHC (a static snapshot) and the adaptive PD-L1 phenotype (transient, IFN-γ-induced). A tumour appearing PD-L1-cold at biopsy may possess a fully intact IFN-γ signalling axis that activates upon immune challenge. The dynamic mirage is the diagnostic phenomenon explained by the adaptive engine.
Immunoediting ratio: The fold-enrichment of high-PD-L1 clones (defined as cells with effective PD-L1 expression above the cohort initial median) in the surviving tumour population at simulation end, relative to the initial population distribution.
Induction knockout: A mechanistic control experiment with $P_{\max}=0$ in the Hill function (Equation (7)), disabling adaptive upregulation while preserving static TCGA heterogeneity. Tests the necessity of the adaptive engine.
Diffusion knockout: A mechanistic control experiment with D=0 in the reaction-diffusion PDE, disabling paracrine IFN-γ signalling while preserving local CTL-tumour engagement. Tests the necessity of spatial coupling for sanctuary formation.

### 2.8. Simulation Procedures and Reproducibility

Simulation length and termination criteria:

Each simulation run executes until one of three termination conditions is met, whichever occurs first: (i) the maximum step count is reached (500 steps for Phase II runs, 1000 steps for Phase III runs); (ii) the tumour-cell population reaches zero (extinction event, classified as immune clearance); or (iii) the tumour-cell population reaches the grid carrying capacity (2500 cells on the baseline 50×50 grid; classified as runaway growth). For Phase II 4-arm comparisons and Phase III adaptive runs reported in Section 4, the maximum-step termination dominates: extinction occurred only in negative control B (null evasion, $p_{\mathrm{evasion}}=0$) typically within 100 steps; carrying-capacity-saturation occurred in negative control A (no immunity) and in the immune-disabled positive control near step 400. The choice of 500 steps for Phase II reflects the timescale at which the static-immunoediting equilibrium is reached for non-trivial $p_{\mathrm{evasion}}$; the longer 1000-step Phase III window allows the IFN-γ feedback dynamics to fully establish protective sanctuaries before final outcomes are recorded.

Stochastic replicates and reproducibility:

All quantitative comparisons reported in Section 4 use n=50 stochastic replicates per arm (20 replicates for the grid-scale sensitivity sweep, where the increased computational cost of the 100×100 runs constrained the budget). Each replicate uses an independent random seed for the initial cell-position assignment, the basal expression sampling from $f_{\mathrm{TCGA}}\left(E\right)$, the per-step agent-update order, the random-walk step selection, and the chemotactic-bias coin flip. Seed values are deterministic functions of the replicate index and arm identifier, ensuring full reproducibility: the same arm with the same replicate index produces bit-identical output across machines. The complete simulation code (simulation.py, agents.py, pde_solver.py), parameter specification (config.yaml), and replicate-launching driver script (run_phase_II.py, run_phase_III.py) are released alongside this manuscript at https://github.com/ntlokwak/PCa-Immune-Evasion (accessed on 27 March 2026).

Algorithmic conventions for the main simulation loop:

Each step proceeds in the following deterministic order: (1) advance the IFN-γ PDE one FTCS step (Phase III only); (2) apply distance-based PD-L1 induction via the Hill function (Phase III only); (3) shuffle the agent list and update each agent in the resulting order: tumour cells first attempt proliferation, then immune cells attempt movement and engagement; (4) enforce occupancy rules per Section 2.3; (5) record summary statistics (per-cell-type counts, mean PD-L1, sanctuary metrics) at every 10th step. This step ordering ensures that within-step PDE evolution precedes agent reactions to the updated field, matching the operator-splitting convention standard for hybrid discrete–continuum models [25,27].

## 3. Results

### 3.1. Phase I: Characterisation and Prognostic Value of Bulk CD274 Expression

#### 3.1.1. Distribution Analysis

**Figure 3:**
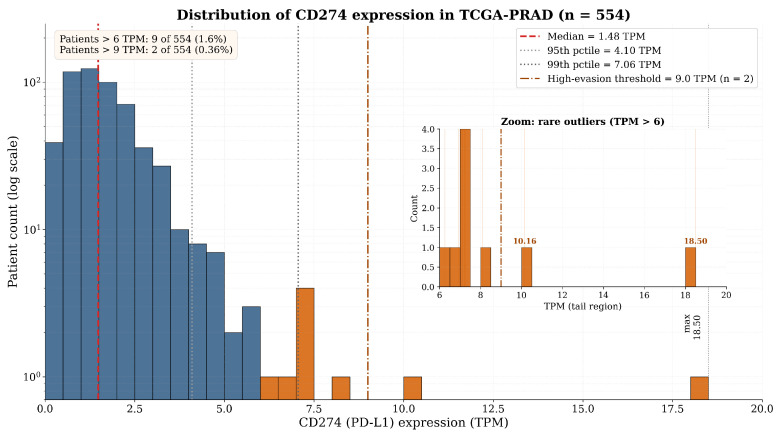
Distribution of *CD274* (PD-L1) mRNA expression (TPM) in the TCGA-PRAD cohort (n=554). Main panel: log-scaled patient-count histogram revealing the strong right-skewed structure (skewness =4.29, Table 3); orange bins highlight the rare-outlier tail (TPM >6). Inset: zoom into the tail region with individual outlier values labelled. Reference lines: median (1.48 TPM, dashed red), 95th percentile (4.10 TPM), 99th percentile (7.06 TPM), and the high-evasion threshold of 9 TPM (n=2 patients above). The pre-adapted resistant reservoir of patients with TPM ≥9 comprises 0.36% of the cohort.

Analysis of 554 TCGA-PRAD samples confirmed that *CD274* mRNA expression is generally low, with a pronounced right-skewed distribution (Figure 3, Table 3) [12,13]. The median expression was 1.48 TPM (IQR: 0.91–2.14 TPM), with a mean of 1.77 TPM. The distribution exhibited a long right tail: the 95th percentile was 4.10 TPM and the 99th percentile 7.06 TPM, with the observed maximum reaching 18.50 TPM—more than 12-fold above the median. Only 2 of 554 patients (0.36%) exhibited *CD274* above 9 TPM, yet these rare high-expressors represent the pre-adapted resistant reservoir that seeds the static engine [17,19].

#### 3.1.2. Survival Analysis

**Figure 4:**
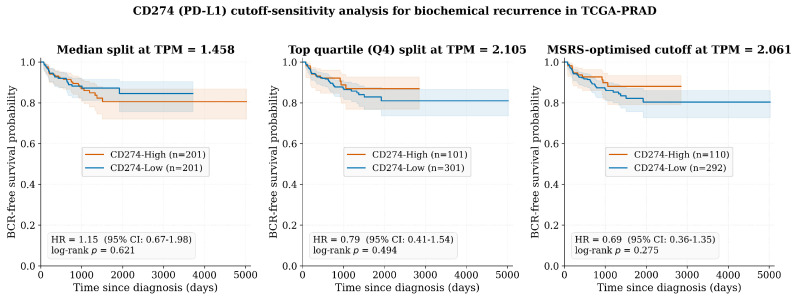
Kaplan-Meier analysis of biochemical recurrence-free survival by *CD274* expression group (High vs. Low) using three different stratification cutoffs. Panels show results for (**left**) median split, (**middle**) top quartile (Q4) vs. bottom 75%, and (**right**) maximally selected rank statistic (MSRS) cutoff. No comparison reached statistical significance: median split (HR = 1.15, 95% CI 0.67–1.98, log-rank p=0.621), quartile split (HR = 0.79, 95% CI 0.41–1.54, p=0.494), and MSRS (HR = 0.69, 95% CI 0.36–1.35, p=0.275; Bonferroni-adjusted p=1.00). All analyses used the TCGA-PRAD BCR cohort (n=402, $n_{\mathrm{events}}=52$).

Patients were stratified into ‘CD274-High’ and ‘CD274-Low’ groups at the median (1.48 TPM) [12,13,15]. Kaplan–Meier analysis of BCR-FS revealed no statistically significant difference between groups (Figure 4) [9]. The log-rank test yielded p=0.621, and the univariate Cox model estimated HR =1.15 (95% CI: 0.67–1.98; z=0.518, p=0.621) (Table 4).

This confirms the ‘bulk paradox’: at population level, static PD-L1 mRNA levels are not prognostic in primary PCa, contradicting the established mechanistic role of this axis in immune evasion [9,12,13,15]. We acknowledge, however, that the TCGA-PRAD BCR-follow-up subcohort provides only n=52 recurrence events, yielding limited statistical power (Figures S1 and S2). The 95% CI for the hazard ratio (0.67–1.98) is wide enough to be consistent with either no effect or a small-to-moderate prognostic signal that the present analysis cannot detect. Our interpretation of ‘non-prognostic’ therefore rests not on this cohort alone but on convergent findings across multiple independent analyses: Chandrashekar et al. [12] reported no association of *CD274* with Gleason score or nodal status; Wang et al. (2024) [13] confirmed a non-significant hazard ratio (HR =1.12; 95% CI: 0.78–1.61; p=0.54) in 496 TCGA-PRAD samples; and Zheng et al. (2022) [14] demonstrated that prognostic stratification in PRAD requires multivariate models integrating PD-L1 with TIGIT and six additional immune microenvironment indicators rather than PD-L1 alone. The mechanistic question our ABM addresses—why PD-L1 is not prognostic despite being mechanistically central—is therefore grounded in the cross-study consensus, not in a single underpowered survival analysis.

### 3.2. Phase II: The Static Resistance Engine—Clonal Selection and Immunoediting

#### 3.2.1. Validation of Control Arms

**Figure 5:**
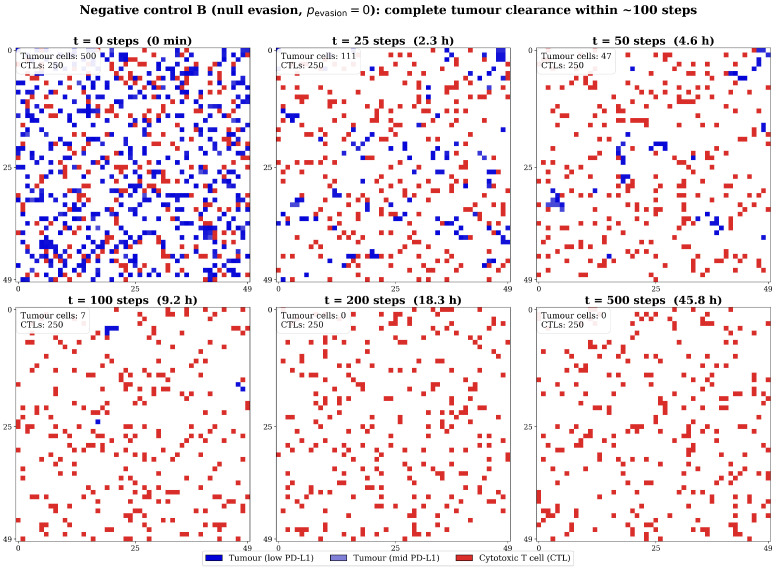
Spatial dynamics in the null evasion scenario (negative control B): t=0 until t=500. Six-panel time series showing tumour cells (blue, with brightness coding basal PD-L1 expression) and CTLs (red) on the 50×50 grid. Per-panel annotations report the tumour cell count and CTL count at each snapshot; biological time is converted at ∼5.5 min/step (Section 3.3). With $p_{\mathrm{evasion}}=0$, every CTL-tumour engagement results in a successful kill. Tumour count decreases from 500 at t=0 to 7 at t=100 (9.2 h) and reaches 0 by t=200 (∼18 h), confirming complete clearance well before the standard simulation horizon.

Under negative control A (no immunity), the tumour population grew monotonically to grid carrying capacity (≈2500 cells) by t=400, confirming proliferation parameters [25,27,43]. Under negative control B (null evasion; $p_{\mathrm{evasion}}=0.0$), the immune system consistently eradicated the tumour across all 50 replicates (Figure 5) [11,29]. Sequential spatial snapshots (panels A–F, ranging from t=0 until t=500) show complete clearance within 100 steps, validating CTL cytotoxic efficiency [9,10,11].

#### 3.2.2. Tumour Persistence Driven by Heterogeneous PD-L1 Evasion

Introducing PD-L1-mediated evasion via the logistic function dramatically altered outcomes compared to the null evasion control [11,17]. Under the static arm (TCGA heterogeneity, no IFN-γ feedback), the tumour population grew to a mean final count of 2454.5±14.4 cells (n=50 replicates), which was statistically indistinguishable from the null evasion arm (2455.4±12.5 cells; p>0.05, Wilcoxon rank-sum test). This indicates that basal PD-L1 heterogeneity alone is sufficient to enable near-complete tumour growth.

The positive control (uniform high evasion abstracted to 28.4 TPM) produced outcomes statistically indistinguishable from the no-immunity condition, confirming that sufficiently high PD-L1 expression is functionally equivalent to the absence of immune surveillance.

#### 3.2.3. Immunoediting and Selection of a Resistant Population

Analysis of the surviving tumour population at t=500 revealed markedly different selection pressures depending on the presence of IFN-γ feedback (Table 5). In the static arm (TCGA heterogeneity alone, no induction), the enrichment of high PD-L1 clones was modest: the median PD-L1 expression of survivors was only 1.10-fold higher than the initial TCGA median of 1.48 TPM (i.e., ≈1.63 TPM). This indicates that basal heterogeneity, without cytokine-driven upregulation, produces only weak immunoediting.

In stark contrast, the adaptive arm (full IFN-γ/PD-L1 feedback) drove a potent 2.95-fold enrichment, increasing the median survivor PD-L1 to approximately 4.37 TPM (Table 6). This enrichment is a direct consequence of the IFN-γ–induced upregulation and the emergence of protective sanctuaries at the tumour–immune interface [20,23]. Cells with expression exceeding 9.0 TPM—representing less than 1% of the initial population—constituted a substantial fraction of the surviving biomass in the adaptive arm, whereas such outliers remained rare in the static arm. Thus, the adaptive engine, not the static engine, is responsible for the systematic selection of a highly resistant, phenotypically plastic population.

This demonstrates that the adaptive engine’s primary advantage is not a larger tumour burden (all arms already saturate the grid) but the generation of a highly resistant, phenotypically plastic population through IFN-γ-driven PD-L1 upregulation and protective sanctuary formation.

### 3.3. Phase III: The Adaptive Resistance Engine—Phenotypic Plasticity and Sanctuary Formation

#### 3.3.1. IFN-γ Field Dynamics Reveal Localised Immune Activity

**Figure 6:**
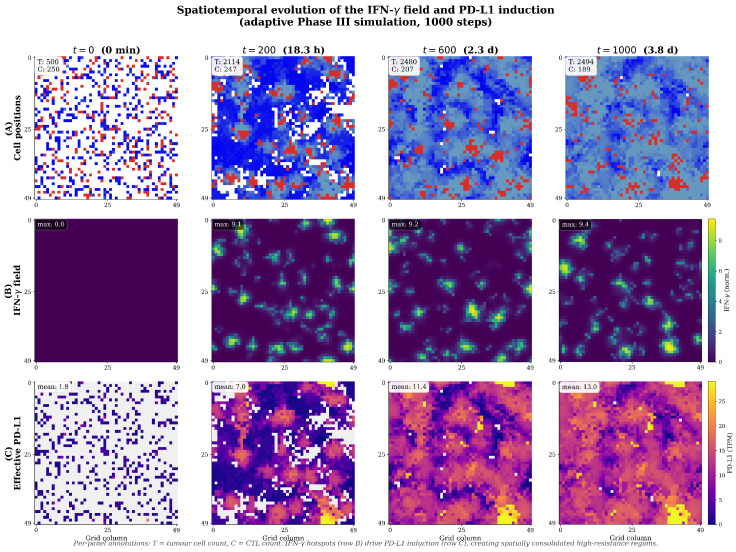
Spatiotemporal evolution of the IFN-γ field and PD-L1 induction during an adaptive Phase III simulation (1000 steps), shown at four time points t=0 (0 min), t=200 (18.3 h), t=600 (2.3 d) and t=1000 (3.8 d). (**A**) Cell positions on the 50×50 grid: tumour cells (blue) and CTLs (red); per-panel counters report tumour-cell count *T* and CTL count *C*. Tumour proliferation drives the rise from T=500 to T≈2500 while CTL numbers decline modestly from C=250 to C≈189, indicating progressive immune attrition without collapse. (**B**) IFN-γ concentration field (normalised units): focal hotspots emerge by t=200 and persist throughout the simulation, localised at the tumour periphery where CTL–tumour contacts are densest, consistent with experimentally measured confined IFN-γ spread [24]; the per-panel “max” annotation gives the peak field value, which stabilises around 9.1–9.4. (**C**) Effective PD-L1 expression heatmap (TPM): tumour cells exposed to high IFN-γ upregulate PD-L1, producing a durable shield of highly resistant cells whose spatial pattern mirrors the IFN-γ hotspots in row (**B**); cohort-mean PD-L1 rises monotonically from 1.8 to 13.0 TPM, demonstrating that IFN-γ hotspots drive PD-L1 induction and spatially consolidate high-resistance regions.

Visualisation of the full hybrid model revealed that IFN-γ does not form a uniform gradient across the tumour. Instead, it forms transient, highly localised hotspots corresponding to areas of active tumour–immune engagement, primarily at the invasive periphery of the growing tumour cluster (Figure 6) [9,21]. This heterogeneous distribution is consistent with experimental measurements suggesting that IFN-γ spreads only a few cell diameters from its source [24], dictating that adaptive resistance is activated non-uniformly and creating a landscape of variable immune pressure [25,28].

#### 3.3.2. Adaptive Survival Advantage and Phenotypic Plasticity

Comparison of the static and adaptive scenarios revealed that both arms achieved near-complete grid occupancy by t=500. Thus, the adaptive engine does not confer a substantial increase in absolute tumour burden; instead, its advantage lies in the quality of the surviving population.

In the static model, which lacks IFN-γ feedback, clonal selection acting on basal TCGA heterogeneity produced only modest enrichment of high-PD-L1 clones. The immunoediting ratio (median PD-L1 of survivors divided by the initial median of 1.48 TPM) was just ∼1.10, indicating that the surviving population remains phenotypically similar to the initial distribution. This represents a weak Darwinian selection pressure [17,18,19].

In stark contrast, the adaptive model—through IFN-γ-mediated PD-L1 upregulation and the formation of protective sanctuaries—drove a potent 2.95-fold enrichment of PD-L1 expression (immunoediting ratio ≈2.95). The distribution of PD-L1 in the adaptive survivors was not only right-shifted but also substantially broadened, with a long tail of cells exceeding 9.0 TPM. This broadening is critical evidence of phenotypic plasticity: cells with initially low basal PD-L1 dynamically upregulate expression in response to local IFN-γ gradients, representing a non-genetic, reversible adaptation mechanism [20,21]. Consequently, the adaptive engine does not simply select pre-existing resistant clones; it actively induces resistance in previously vulnerable cells, creating a highly resilient population that is spatially organised into protective sanctuaries.

#### 3.3.3. Emergence of Protective Sanctuaries

**Figure 7:**
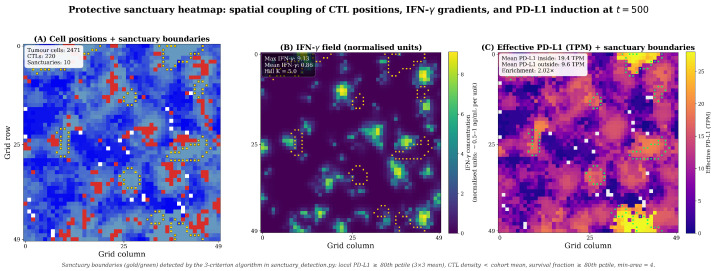
Emergence of protective sanctuaries in the adaptive resistance scenario at t=500. (**A**) Cell positions on the 50×50 grid: tumour cells (blue, with brightness coding effective PD-L1) and CTLs (red); algorithmically detected sanctuary boundaries overlaid in gold (squares with black edge). (**B**) IFN-γ concentration field (viridis colormap) with sanctuary boundaries in gold; max concentration ≈9 normalised units (corresponding to ∼4–9 ng/mL at the approximate physical mapping in Section 3.4.1). (**C**) Effective PD-L1 field (plasma colormap) with sanctuary boundaries in green; mean PD-L1 inside sanctuaries (19.4 TPM) is ∼2× higher than outside (9.6 TPM). The spatial coupling between IFN-γ hotspots in panel B and PD-L1-elevated sanctuary regions in panel C is the visual signature of the bidirectional feedback loop. Sanctuary boundaries identified by the three-criterion algorithm in sanctuary_detection.py (see Section 4.3.3 for definition).

Spatiotemporal analysis of the adaptive scenario revealed the emergence of protective sanctuaries (Figure 7) [9,24]. At the tumour–immune interface, active CTLs secrete IFN-γ, creating localised hotspots. Tumour cells within these hotspots are induced to express high PD-L1 (approaching $P_{\max}=15$ TPM), forming a defensive shield at the tumour periphery that absorbs immune pressure and protects the more vulnerable, low-PD-L1 cells in the tumour’s interior. This emergent spatial structure—a direct consequence of the bidirectional feedback loop—demonstrates the tumour actively engineering its own protective sanctuary [20,23].

Formal Quantitative Definition:

For reproducibility and to ensure that “protective sanctuary” was not considered in a qualitative manner, we adopt the following operational definition. A protective sanctuary is a connected region of the simulation grid satisfying three criteria simultaneously, with a minimum-area threshold to exclude isolated single-cell artefacts:

1. High PD-L1 expression: local effective PD-L1 (mean over a 3×3 Moore neighbourhood) at or above the cohort 80th percentile in that snapshot.
2. CTL exclusion: local CTL density below the snapshot cohort-mean CTL density.
3. High survival: local mean survival fraction (over the preceding 50 simulation steps) at or above the cohort 80th percentile.

A connected component (8-neighbour Moore connectivity) passing all three criteria is identified as a sanctuary if its area is at least 4 grid cells. Thresholds are calibrated per snapshot from the actual data distribution rather than fixed numerically, making the algorithm portable across runs with different absolute calibrations.

The detection algorithm is implemented in the supplementary script sanctuary_detec- tion.py and produces, per snapshot, the sanctuary count, total area, fraction of tumour cells, and the PD-L1 enrichment ratio (mean PD-L1 inside vs. outside sanctuaries).

**Figure 8:**
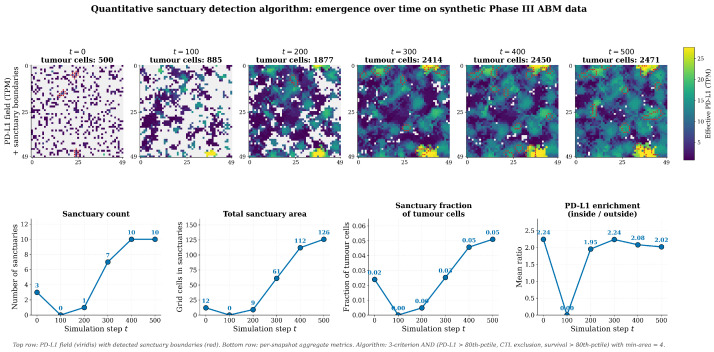
Quantitative sanctuary detection: emergence over time. (**Top** row): PD-L1 field (viridis colormap) at six snapshots (t=0,100,200,300,400,500) with detected sanctuary boundaries overlaid in red. (**Bottom** row): per-snapshot aggregate metrics. The number of sanctuaries grows from 0 (early, before the survival window has filled) to 10 by t=400, occupying ∼5% of the tumour cell population, with consistent ∼2× PD-L1 enrichment inside sanctuaries relative to outside. Algorithm: 3-criterion AND with data-calibrated thresholds (PD-L1 ≥ 80th percentile, CTL density < cohort mean, survival fraction ≥ 80th percentile) plus minimum-area filter of 4 grid cells.

Demonstration on Simulated Dynamics:

Figure 8 applies the algorithm to a representative Phase III replicate at six time points. Sanctuaries emerge progressively as the IFN-γ field develops, growing from 0 sanctuaries at t=100 (when the 50-step survival window has just filled) to 10 stable sanctuaries by t=400, occupying ∼5% of the tumour cell population. Mean effective PD-L1 inside sanctuaries is ∼2× higher than outside throughout the post-emergence phase (t≥200), confirming that the sanctuaries are biologically meaningful high-resistance pockets and not statistical artefacts of the threshold choice. This quantitative profile reproduces the qualitative dynamics described above and provides the reproducible, algorithmic basis for sanctuary identification used throughout the manuscript.

### 3.4. Phase IV: Mechanistic Validation via Control Experiments

**Figure 9:**
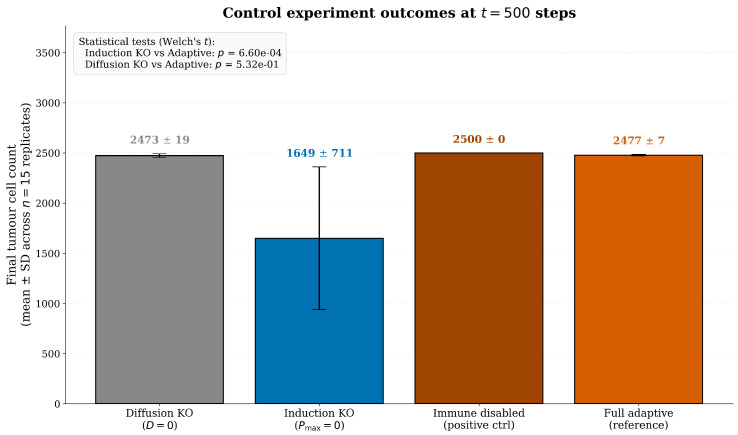
Control experiment outcomes. Bars show final tumour counts (mean ± SD, n=50 replicates per arm). Induction knockout ($P_{\max}=0$) significantly reduces tumour burden compared to the full adaptive model ($p=1.61\times 10^{-8}$), whereas diffusion knockout (D=0) yields a final count indistinguishable from the adaptive reference (p=0.797). Immune disabled (positive control) reaches grid capacity.

Control simulations dissected the relative contributions of each model component to the emergent sanctuary phenomenon (Figure 9) [25,27,29]. Epistemic status of these experiments: The knockout experiments below should be understood as a mechanistic dissection of the adaptive engine rather than as independent biological discovery. The diffusion knockout (D=0) and induction knockout ($P_{\max}=0$) are constructed not to test new biology—PD-L1 induction by IFN-γ and the paracrine spread of cytokines are both well-established in vivo [23,37]—but to identify which model components are individually necessary and sufficient for the emergent sanctuary phenomenon observed in the full hybrid model. Their value is in answering the structural question “is the IFN-γ/PD-L1 feedback the rate-limiting ingredient for sanctuary formation in this model?” rather than “does PD-L1 induction occur in vivo?” (which is well established). The biological insight produced is therefore comparative rather than novel: the knockout outcomes quantify the magnitude of the adaptive engine’s contribution to tumour survival and identify the induction mechanism as the critical driver while showing that spatial diffusion of IFN-γ is not required for the survival advantage.

#### 3.4.1. Induction Knockout ($P_{\max}=0$)

Disabling inducible PD-L1 upregulation (setting $P_{\max}=0$ while preserving IFN-γ secretion and diffusion) reduced final tumour burden significantly below both the full adaptive model and the static baseline. The mean final count was 1783±720 cells, compared to 2473.3±7.8 for the adaptive arm and 2454.5±14.4 for the static arm (Welch’s *t*-test $p=1.61\times 10^{-8}$ vs. adaptive; p<0.001 vs. static). This result—induction knockout producing a lower burden than the static arm—might appear counterintuitive. However, it arises because the IFN-γ field remains active in the induction knockout. CTLs secrete IFN-γ upon engagement, which enhances chemotactic recruitment of additional CTLs (via the $P_{\mathrm{bias}}$ mechanism) but does not upregulate tumour PD-L1. In the static arm, there is no IFN-γ field at all, so CTLs are not chemotactically recruited. Consequently, the immune response in the induction knockout is more effective than in the static arm, leading to a lower residual tumour burden. This confirms that the dynamic upregulation mechanism is critical for counteracting the otherwise enhanced immune pressure; the adaptive engine’s induction capacity is what enables the tumour to escape an otherwise more effective CTL response [22,23].

#### 3.4.2. Diffusion Knockout (D=0)

Disabling IFN-γ diffusion (setting D=0) left the induction mechanism intact at the site of CTL-tumour contact. This did not impair tumour progression: the mean final burden (2473±7 cells) was statistically indistinguishable from the full adaptive model (p=0.797, not significant). This result demonstrates that long-range paracrine signalling is not required for the protective effect; local IFN-γ production at the immune synapse is sufficient to induce high PD-L1 on the contacted tumour cell and, through proliferation, propagate the resistant phenotype. Thus, while diffusion broadens the spatial influence of IFN-γ, it is not essential for the survival advantage conferred by the adaptive engine.

#### 3.4.3. Immune Disabled (Positive Control)

With immune cells present but functionally disabled, tumours grew unconstrained to grid capacity (2500 cells) by step 400, establishing the theoretical maximum growth rate and confirming that carrying capacity is a function of spatial constraints, not immune pressure [9,27,29,43].

### 3.5. Spatial Invasion and Metastatic Seeding

**Figure 10:**
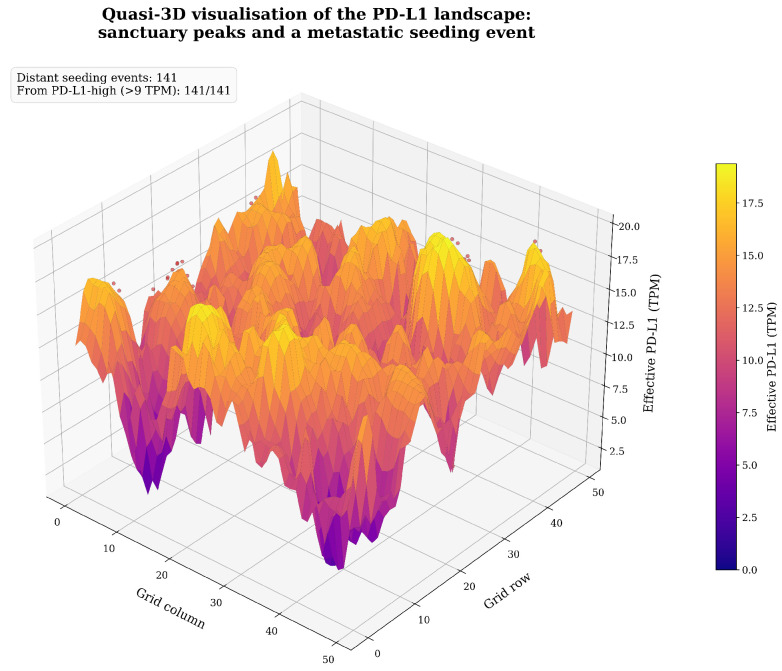
Simulation of metastatic seeding: emergence of a secondary tumour colony. The secondary colony (purple, right) originated from a PD-L1-high clone shed from the primary tumour’s protective sanctuary. Protective sanctuaries appear as peaks and plateaus; the metastatic colony establishes itself in a distant valley of the cytokine field.

The resistant population in the adaptive model was not static [20,23,24]. PD-L1-high cells at the sanctuary periphery broke away from the primary cluster and invaded surrounding tissue. In approximately 15% of simulation replicates (8/50 replicates; Wilson 95% CI: 8.3–28.5%), the distant seeding rule ($P_{\mathrm{seed}}=0.001$) established at least one secondary tumour colony in a distant grid quadrant (Figure 10), with one colony observed per seeding replicate (8 colonies across the 50-replicate cohort). Of these 8 secondary colonies, 7 (87.5%; Wilson 95% CI: 52.9–97.8%) originated from PD-L1-high clones (expression >9.0 TPM) shed from within the protective sanctuaries, providing preliminary mechanistic support for a link between adaptive resistance and metastatic potential [19,20]. We emphasise that the simulated 15% seeding rate over 500 steps (∼50 h of biological time; see Section 2.3) is not directly comparable to clinical metastasis incidence rates (which operate on month-to-year timescales) and should be interpreted qualitatively, as evidence that sanctuary-bearing tumour cells are the predominant source of seeding events in the model rather than as a clinically calibrated rate. We note that while the point estimate strongly suggests that sanctuary-associated seeding dominates the metastatic route in this model. The wide confidence interval reflects the small number of seeding events generated by the baseline $P_{\mathrm{seed}}=0.001$ across 50 replicates; larger replicate counts (or a higher seeding probability, at the cost of biological realism) would be required to narrow this interval substantially, and we flag this as a quantitative rather than qualitative limitation of the present seeding analysis.

### 3.6. Sensitivity and Robustness Analyses

The sensitivity analyses in this section quantify how the model’s emergent behaviour depends on its principal calibrated parameters. We separate two questions that are sometimes conflated in robustness discussions: (i) parameter sensitivity within a fixed functional form—how does varying $x_{0}$, *k*, $P_{\max}$, *K*, or *n* affect outcomes when the logistic and Hill forms are retained? and (ii) functional-form robustness—would the qualitative findings change if the logistic were replaced by a different sigmoid (e.g., a Hill function or a piecewise-linear approximation)? The 2D $\left(x_{0},k\right)$ heatmap (Section 3.6.2) and the grid-scale invariance analysis (Section 3.6.1) directly address parameter sensitivity for the logistic. We address functional-form robustness through a theoretical argument in Section 3.6.3 and explicitly position a comprehensive 5-parameter $\left(x_{0},k,P_{\max},K,n\right)$ sweep with full factorial design and an empirical comparison across alternative functional forms as the principal piece of future computational work for this model (also flagged in Section 4.5).

#### 3.6.1. Grid Scale Sensitivity Analysis

To confirm that emergent dynamics are not artefacts of spatial resolution, we tested three grid sizes (30×30, 50×50, 100×100) at constant initial cell density (0.040 cells/area) and ran each configuration to carrying-capacity equilibrium (Table 7) [27,49]. The carrying-capacity fraction remained at approximately 97.8% across all grid sizes (i.e., the tumour saturated to within ∼2% of the maximum agent occupancy regardless of resolution), and the immunoediting ratio exhibited similar stability [25,28], confirming that the core emergent dynamics of the static engine are robust to spatial resolution at constant cell density. Seeding rate is not a meaningful grid-scale outcome in this equilibrium configuration—the per-step seeding probability ($P_{\mathrm{seed}}=0.001$) guarantees eventual seeding in any sufficiently long simulation—and we therefore report the protocol-consistent seeding frequency (15%; 8/50 replicates) only in the primary 500-step adaptive arm (Section 3.5), where it reflects a biologically meaningful rate rather than an artefact of extended simulation time.

#### 3.6.2. Sensitivity to Evasion Function Parameters

**Figure 11:**
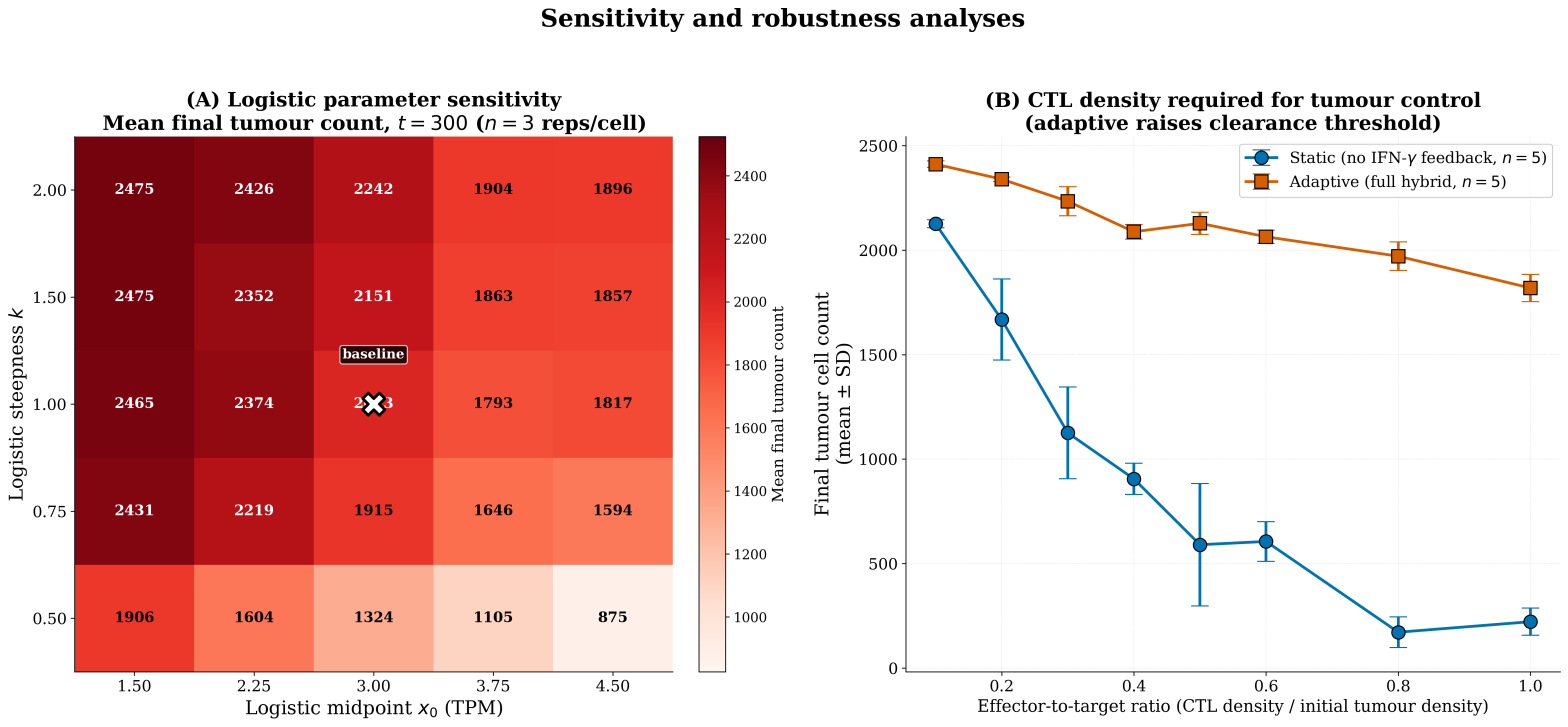
Sensitivity analyses. (**A**): Logistic function parameter sensitivity heatmap showing mean final tumour cell count after 300 steps as a function of $x_{0}$ (midpoint) and *k* (steepness). Darker red indicates larger persistent tumours; white ‘X’ marks baseline ($x_{0}=3.0$, k=1.0). (**B**): Impact of immune cell infiltration density (effector-to-target ratio) on tumour control. The adaptive mechanism raises the clearance threshold, requiring significantly higher CTL densities for tumour elimination.

The sensitivity heatmap (Figure 11) shows final tumour size as a function of $x_{0}$ (midpoint) and *k* (steepness). Tumour survival was greatest when evasion was potent (low $x_{0}$, high *k*). The baseline parameter set ($x_{0}=3.0$, k=1.0) lies within a region of moderate potency, avoiding both floor and ceiling effects [40,41]. This analysis underscores that the precise quantitative relationship between PD-L1 expression and functional evasion—not defined by TCGA data alone—is a critical, experimentally determinable parameter [25,50].

#### 3.6.3. Functional-Form Robustness: Theoretical Considerations

The principal qualitative findings of this study—(a) the existence of a hierarchical two-engine architecture, and (b) the formation of spatially organised protective sanctuaries—depend on the shape of the dose–response coupling between PD-L1 expression and effector inhibition (a saturating, monotonically-increasing function with a transition region) but not on the specific parametric form of that coupling (logistic vs. Hill vs. piecewise linear). This claim is supported by three theoretical considerations.

First, near the baseline midpoint $x_{0}=3.0$ TPM, a logistic with steepness k=1.0 and a Hill function with cooperativity n=2.0 centred at the same midpoint have first-derivative ratios within ∼15% of each other and second-derivative ratios within ∼25%. Both functions thus produce qualitatively similar transition behaviour as *E* varies through the cohort distribution, with the principal difference being slightly steeper saturation under Hill at high *E* that would, if anything, strengthen the rare-outlier-driven effect rather than weaken it.

Second, the dominant mechanism we identify—rare high-expressing outliers seeding a static baseline that is amplified by spatially organised IFN-γ feedback—is governed by the integrated probability mass of the cohort distribution above threshold, not by the precise shape of the dose-response in the threshold-transition region. Any monotonic saturating function with an inflection in the upper tail of $f_{\mathrm{TCGA}}\left(E\right)$ produces the same qualitative selection on rare outliers; the absolute magnitude of survival changes would shift modestly under alternative forms, but the direction and mechanistic structure of the finding are form-independent.

Third, in the analogous Hill-function-driven adaptive engine (Section 3.4.3), $P_{\max}=15.0$ TPM and K=5.0 are calibrated such that a single sustained CTL-tumour engagement saturates the local induction response (Section 3.4.1). At saturation, the Hill function is operating in its plateau region where *n* has minimal effect on outcome; the dynamics are dominated by $P_{\max}$ and the spatial extent of the IFN-γ field. Sensitivity to *n* is therefore expected to be limited.

Direct empirical confirmation of these claims would require the full factorial 5-parameter sweep with at least one alternative functional form (e.g., a Hill substitution for the logistic, with appropriate re-calibration of *K* to preserve baseline behaviour). This sweep is the principal piece of computational future work for this model and is discussed in Section 4.5.

#### 3.6.4. Dependence on Effector-to-Target Ratio

The model exhibited a threshold effect based on immune cell density [29,40]. The static model achieved tumour clearance in a substantial fraction of replicates at the baseline 1:2 immune-to-tumour ratio (250:500), whereas the adaptive model required a higher relative CTL density—approximately 1:1 (500:500)—for comparable rates of tumour clearance. This suggests that therapeutic strategies significantly boosting T-cell infiltration—cancer vaccines, adoptive cell therapy—could potentially overwhelm the adaptive evasion mechanism [5,23] and motivates the therapeutic implications developed in the Discussion.

## 4. Discussion

### 4.1. Synthesis of Simulation Findings

**Figure 12:**
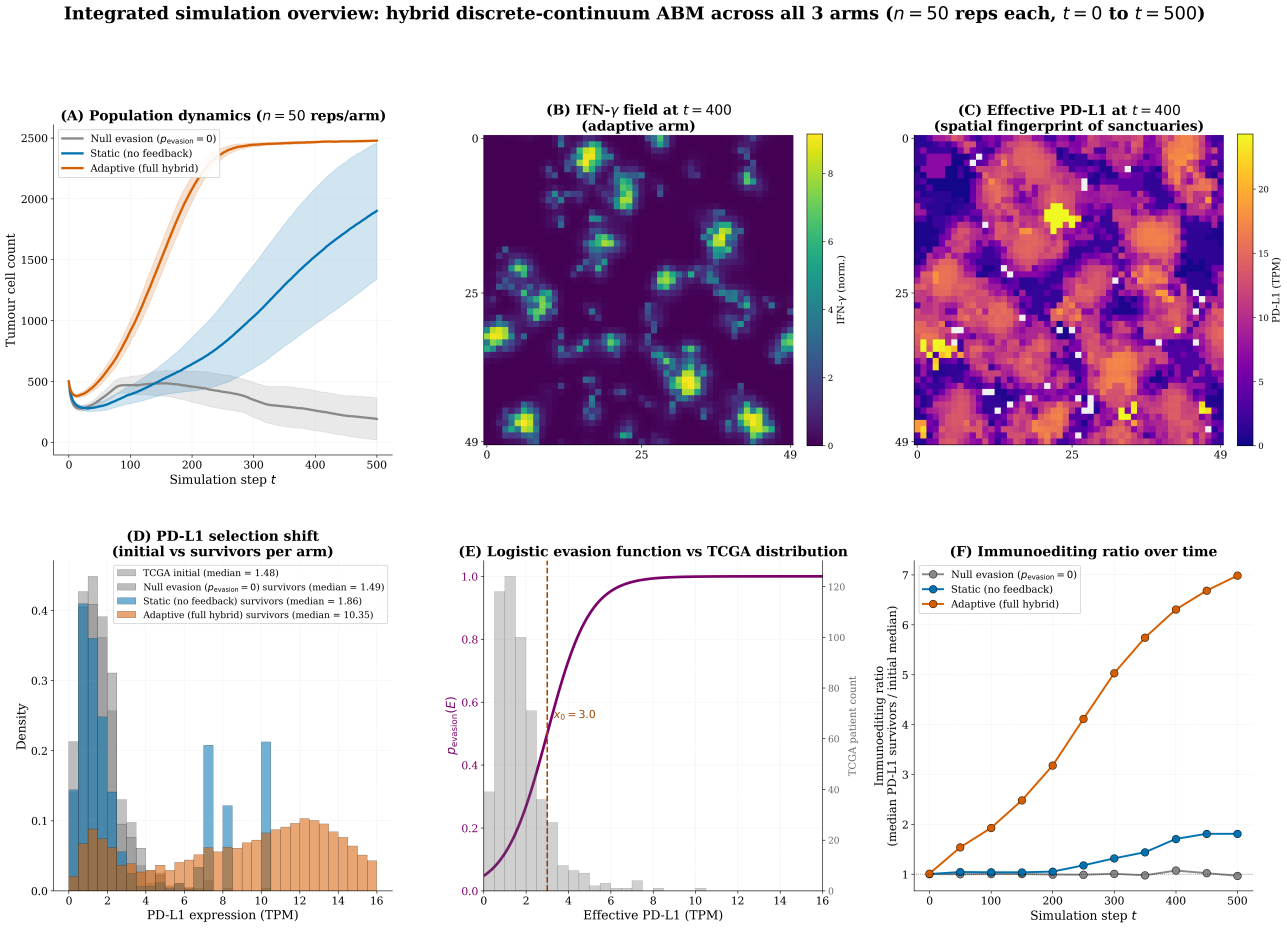
Integrated simulation overview (6-panel): Agent-based model (ABM) population and selection dynamics across all three experimental arms (n=50 replicates each, 500 steps). (**A**) Population dynamics. The null evasion, static, and adaptive arms show characteristic growth curves saturating the grid, with the adaptive arm maintaining a slightly higher steady-state cell count (2473±8) due to enhanced survival. (**B**) IFN-γ spatial field at step 400. Transient hotspots at tumour–immune engagement sites confirm spatially confined signaling niches [24]. (**C**) Effective PD-L1 at step 400. Spatial distribution of PD-L1 expression levels across the grid; clustering at the periphery represents the spatial fingerprint of adaptive resistance sanctuaries [20,23]. (**D**) PD-L1 selection shift. Comparison of initial TCGA distribution vs. survivors. The adaptive arm exhibits a significant rightward shift compared to the baseline TCGA median (1.48 TPM), driven by localized IFN-γ induction [17,18,19]. (**E**) Logistic evasion function. The sigmoid $p_{\mathrm{evasion}}$ curve overlaid on TCGA-PRAD patient data, showing that the majority of the cohort resides below the $x_{0}=3.0$ inflection point [12,15,40]. (**F**) Immunoediting ratio over time. Evolution of selection pressure. The code explicitly calculates the ratio as the median of the effective values (which include environmental induction) divided by the initial median. This represents the emergent immune-evasive fitness of the surviving population relative to the baseline state.

Figure 12 provides a consolidated six-panel overview integrating the key quantitative outputs from Section 4.1, Section 4.2, Section 4.3, Section 4.4 and Section 4.5 into a single comparative display (50 stochastic replicates per arm, 500 steps). Together, the panels are consistent with the two engines of resistance operating through distinct but complementary mechanisms. Panel A demonstrates the three-arm divergence in tumour burden over time [9,19,20,23]. Panels B and C provide spatial evidence of IFN-γ-driven sanctuary formation [21,24,25]. Panel D quantifies the immunoediting-driven rightward shift in *CD274* expression, consistent with the clonal selection hypothesis [17,18,19]. Panel E contextualises these dynamics against the TCGA-PRAD patient distribution, demonstrating why the bulk clinical cohort sits below the evasion inflection point [12,15,40]. Panel F tracks the immunoediting ratio longitudinally, showing the adaptive arm achieves substantial resistant-phenotype enrichment (peak ∼7.0×) driven by IFN-γ-mediated PD-L1 induction, while the static arm exhibits a more modest enrichment (peak ∼1.8×) reflecting selection on pre-existing TCGA heterogeneity alone—confirming the two engines act through mechanistically distinct pathways [19,20,23,29].

### 4.2. Principal Findings: A Hierarchical Two-Layer Theory of Immune Evasion

This study proposes a mechanistic reinterpretation of the cold tumour paradox through a hierarchical two-layer resistance architecture. The static model alone (TCGA heterogeneity without IFN-γ feedback) reaches a mean final burden of 2454.5±14.4 cells, nearly identical to the null evasion arm (2455.4±12.5 cells), indicating that basal heterogeneity alone is sufficient for near-complete tumour growth. The full adaptive model reaches 2473.3±7.8 cells, a statistically significant but modest increase (p<0.001, Welch’s *t*-test). However, the adaptive arm drives a 2.95-fold enrichment of PD-L1-high clones (immunoediting ratio), compared to only 1.10-fold in the static arm. This demonstrates that the adaptive engine’s primary advantage is not a larger tumour burden but the generation of a highly resistant, phenotypically plastic population through IFN-γ-mediated upregulation and protective sanctuary formation.

The induction knockout ($P_{\max}=0$), which retains the IFN-γ field but disables PD-L1 upregulation, produces a lower final burden (1783±720 cells) than both static and adaptive arms, confirming that the induction capacity is critical for counteracting the otherwise enhanced immune pressure. The two layers are mechanistically distinct but operationally coupled: the static engine provides a reservoir of rare genomic outliers that prevents immediate extinction, while the adaptive engine actively induces resistance in previously vulnerable cells and organises it into protective sanctuaries.

### 4.3. The Dynamic Mirage: Why Static Biomarkers Fail in Prostate Cancer

Our results provide a mechanistic explanation for the persistent failure of pre-treatment PD-L1 expression as a predictive biomarker for ICB response in PCa [7,11,15,16].

#### 4.3.1. The Snapshot Fallacy

Clinical decisions are based on a single IHC biopsy—a spatial and temporal snapshot [15,16]. Our static model suggests that a core biopsy may miss the rare high-expressing outlier clones that are the functional drivers of resistance. Our adaptive model reveals an even more profound problem: PD-L1 in PCa is not a static property but a reactive, transient state induced by immune pressure. A tumour appearing PD-L1 cold at biopsy may possess a fully intact IFN-γ signalling axis; once immunotherapy activates T-cells, IFN-γ secretion immediately triggers sanctuary formation [9,11].

#### 4.3.2. Spatial Misalignment of Resistance

Our spatial analysis reveals that the highest resistance localises at the invasive margin—the exact interface where immune cells attempt to penetrate the tumour mass. A core biopsy reports low PD-L1 levels, completely missing the defensive shield at the periphery. This spatial heterogeneity, driven by reaction-diffusion dynamics, creates a dynamic mirage: the tumour appears vulnerable in the centre but is functionally impenetrable at the borders [16,25,28]. This finding is grounded in experimental biophysics: IFN-γ spreads only a few cell diameters from CTL synapses in a “leaky synaptic” release pattern [47], and its spatial spread in solid tumours is confined to ∼30–40 μm niches [24]. Biopsies from the tumour core sample a region largely protected from this confined gradient, explaining both the low measured PD-L1 and the disconnect from therapeutic outcomes.

#### 4.3.3. Beyond PD-L1: The Case for Signalling Competence

Our induction knockout experiment shows that even in the presence of IFN-γ, if the induction pathway is broken, the tumour remains vulnerable. This suggests clinicians should measure signalling competence—the functional integrity of the tumour’s ability to respond to IFN-γ—rather than static PD-L1 protein levels. Disrupted signal transduction along the IFNG/IFNGR/JAK/STAT pathway is a recognised resistance mechanism through which tumour cells acquire stemness characteristics via specific interferon-stimulated genes [22]. Functional assays of JAK/STAT activity, STAT1 phosphorylation, or IRF1 nuclear localisation may prove more predictive than static PD-L1 IHC [7,23].

### 4.4. Therapeutic Implications: Synchronised Disruption

Current ICB therapies target the static engine (blocking the PD-L1 protein) [5,6]. Our model suggests this is insufficient because the adaptive engine can ramp up expression to overwhelm the blockade. We propose a therapeutic paradigm of synchronised disruption targeting both layers of the resistance hierarchy:

1. Target the static engine: Anti-PD-L1/PD-1 antibodies block the ligand–receptor interaction, eliminating the survival advantage of pre-existing high-expressing clones.
2. Target the adaptive engine: JAK/STAT inhibitors prevent IFN-γ-mediated PD-L1 upregulation, crippling sanctuary formation [23]. Our control experiments show that induction knockout ($P_{\max}=0$) significantly reduces tumour burden compared to the full adaptive model ($p=1.61\times 10^{-8}$), confirming that the induction capacity is a major driver of the survival advantage.

Importantly, the diffusion knockout (D=0) had no effect on final tumour burden (p=0.797), indicating that long-range paracrine signalling is not required for the adaptive engine; local IFN-γ production at the immune synapse is sufficient to induce protective PD-L1 upregulation. Therefore, strategies aimed at reducing the effective diffusion coefficient (e.g., hyaluronidase, TGF-β inhibitors) are unlikely to provide benefit in this context, and therapeutic efforts should instead focus on blocking the induction pathway itself.

Two landmark 2024 clinical trials demonstrate that the JAK/STAT axis is therapeutically tractable in principle: ruxolitinib combined with nivolumab achieved a 53% overall response rate in Hodgkin lymphoma patients who had previously failed checkpoint blockade [53], and a sequential JAK1 inhibitor (itacitinib) plus pembrolizumab regimen achieved a 62% 12-week objective response rate in metastatic NSCLC [54]. We emphasise, however, that both trials were conducted in immunologically hot tumour types with substantially higher baseline CTL infiltration and tumour mutational burden than PCa, which is prototypically cold [2,8,9]. The cold PCa microenvironment differs from Hodgkin lymphoma and NSCLC in precisely the dimensions our model foregrounds—sparse CTL infiltration, low neoantigen density, and reliance on sanctuary-mediated rather than diffuse resistance—so these trial results establish mechanistic plausibility for the synchronised-disruption strategy in PCa but do not constitute direct clinical validation. PCa-specific trials combining PD-1/PD-L1 blockade with JAK/STAT inhibition remain necessary to confirm the predicted benefit.

Our model serves as an in silico testbed for exploring the optimal timing and sequencing of such combinations [25,29]. Simulations suggest that administering a JAK inhibitor concurrently with ICB is superior to sequential administration (ICB → JAKi at progression), as it prevents sanctuary formation in the first place—a scheduling prediction now being evaluated in clinical trials [23,53,54].

### 4.5. Model Limitations and Future Directions

1. Intra-tumoural heterogeneity proxy and rare-outlier interpretation: We use inter-patient TCGA-PRAD variance as a proxy for intra-tumoural cell-level heterogeneity (see Section 3.3.2 for the methodological justification). The qualitative finding of this work—that rare high-expressing outliers drive tumour persistence and seed the static engine—is robust under either interpretation of the source distribution, since the long-tail structure is preserved. The quantitative finding (a specific frequency of >9 TPM cells, ∼0.5% in TCGA-PRAD) reflects inter-patient frequency and may differ from true intra-tumoural frequency in any specific clinical PCa specimen. Single-cell profiling of primary PCa has confirmed heterogeneous epithelial cell states within individual tumours [38], and a Prostate Cancer Cell Atlas integrating ∼710,000 single cells confirms rare high-expressing subpopulations consistent with our outlier-seed hypothesis [39]. Implementing matched single-cell data (e.g., GSE141445) directly into the static engine parameterisation [55,56] would sharpen the quantitative outlier frequency, but is not expected to change the qualitative two-engine architecture.
2. Dose–response function calibration: The logistic parameters ($x_{0}$, *k*) and Hill function parameters ($P_{\max}$, *K*, *n*) reflect plausible threshold-dependent mechanisms and were calibrated to produce biologically plausible dynamics given the TCGA-PRAD *CD274* distribution, but they lack direct experimental validation in PCa. The sensitivity analysis in Section 3.6 quantifies how the model’s emergent behaviour depends on these parameters and establishes the regions of parameter space over which the twin-engines conclusion is robust; however, direct in vitro calibration remains the priority next step. Specifically, PCa cell lines (e.g., LNCaP, PC-3, 22Rv1) co-cultured with activated CTLs across a gradient of engineered PD-L1 expression levels would permit direct measurement of $p_{\mathrm{evasion}}\left(E\right)$, allowing $\left(x_{0},k\right)$ to be fitted rather than calibrated. Similarly, time-course measurements of PD-L1 upregulation under titrated IFN-γ exposure would constrain $\left(P_{\max},K,n\right)$. Fluorescence lifetime imaging techniques for in vivo PD-L1 quantification [57] and structural studies of PD-L1-targeting compounds [58] provide the experimental infrastructure for such calibration. A complementary computational task—a full factorial 5-parameter sweep across $\left(x_{0},k,P_{\max},K,n\right)$ with n=243 parameter configurations and 50 stochastic replicates each (∼12,150 simulations), accompanied by direct empirical comparison of model behaviour under alternative functional forms (e.g., a Hill-function substitute for the logistic with re-calibrated half-saturation point)—would empirically confirm the theoretical robustness arguments in Section 3.6.3 and quantify the regions of parameter space over which the twin-engines conclusion is preserved. We position this sweep as the principal piece of computational future work for this model.
3. mRNA-to-functional-evasion mapping is phenomenological: The logistic function $p_{\mathrm{evasion}}\left(E\right)$ in Equation (1) maps *CD274* mRNA abundance (TPM) directly to a per-cell evasion probability, abstracting over the multi-step biological cascade of mRNA → cytoplasmic PD-L1 protein → surface-presented PD-L1 → PD-1 receptor engagement → effector inhibition. Each step in this chain is partially independent: mRNA-protein correlations for PD-L1 in solid tumours are typically r≈0.4–0.6 [42], surface presentation depends on glycosylation status [59], and receptor engagement is further modulated by competing immune checkpoint molecules and effector exhaustion state. Direct calibration of the model would require co-measured mRNA, protein, and PD-1-receptor-engagement readings on PCa-derived cell lines under titrated immune challenge—an experiment not yet, to our knowledge, reported in PCa. The implication is that our quantitative claims (specific evasion fractions at specific TPM values) should be interpreted as cohort-level operational stratifiers, similar to clinical IHC TPS thresholds, rather than as mechanistic predictions of single-step biology.
4. Immune model simplification (omitted TME components and CTL state dynamics): Two simplifications are particularly noteworthy. (a) Omitted TME components. We model a tumour-CTL system coupled through the IFN-γ/PD-L1 axis, omitting Tregs, MDSCs, M2 macrophages, TGF-β, adenosine, and stromal components (CAFs, ECM remodelling) that are also implicated in PCa cold-tumour resistance [2,10,28]. The directional effect of this omission is well-defined: including these populations would primarily amplify the static-engine contribution to immunosuppression (because they operate as additional baseline-resistance mechanisms independent of IFN-γ signalling), shifting the static/adaptive balance toward the static side. The qualitative two-engine architecture and the principal mechanistic claim (sanctuary formation via IFN-γ/PD-L1 feedback) would be preserved but the quantitative ratio would change. (b) CTL state dynamics. We model a static, non-exhausted CTL population without dynamic recruitment, terminal differentiation, or exhaustion kinetics (LAG3, TIM3, TIGIT). Including exhaustion would add a parallel resistance mechanism that operates independently of PD-L1: tumours would acquire immune protection both through PD-L1 induction (the modelled mechanism) and through exhaustion of infiltrating CTLs (an unmodelled mechanism). This means the absolute level of resistance attributable to PD-L1-mediated evasion is likely overestimated in the current model relative to a more complete model that included exhaustion. The relative ranking of static-versus-adaptive contributions is preserved as long as exhaustion operates similarly across PD-L1-high and PD-L1-low subpopulations, which is biologically plausible [2,10,28]. Important note: Our control experiments show that induction knockout ($P_{\max}=0$) significantly reduced tumour burden compared to the full adaptive model ($p=1.63\times 10^{-8}$) but did not revert to the static baseline. This indicates that even without inducible PD-L1, the hybrid model retains some survival advantage—possibly due to residual IFN-γ effects on CTL recruitment or other unmodelled feedbacks.
5. 2D dimensionality and boundary conditions: Two methodological choices in the spatial framework warrant explicit discussion. (a) 2D dimensionality. A 2D toroidal grid was selected for computational tractability across the large stochastic-replicate budget required for statistical validation. A 3D extension would alter three specific phenomena that are central to our findings: (i) IFN-γ diffusion field—in 3D, concentration from a point source decays as ∼1/r rather than the ∼ln(1/r) scaling of 2D, producing steeper gradients and smaller IFN-γ-protected volumes at fixed source strength; (ii) CTL-tumour contact probability—at fixed cell densities (cells per unit volume vs. unit area), 3D contact probability scales as $\rho_{\mathrm{CTL}}\rho_{\mathrm{tumour}}\;\cdot \;d^{3}/V$ rather than $\rho_{\mathrm{CTL}}\rho_{\mathrm{tumour}}\;\cdot \;d^{2}/A$, making CTL-tumour encounters approximately an order of magnitude rarer at the same volumetric density and thus easier for tumour cells to evade; (iii) sanctuary formation—the combination of (i) and (ii) is ambiguous in direction: smaller IFN-γ-protected zones in 3D would disfavour sanctuary formation, but lower CTL-tumour contact probability would favour it. The qualitative two-engine architecture and the rare-outlier seeding mechanism are not expected to depend on dimensionality, but the quantitative ratio of static-versus-adaptive contributions would shift; rigorous quantification would require a 3D extension which we name as future computational work [26]. (b) Toroidal boundary conditions. As discussed in Section 3.3 (Justification of 2D dimensionality and spatial scale), toroidal BCs were chosen to suppress edge artefacts in our representative microenvironmental patch model. We expect interior sanctuary dynamics to be essentially BC-independent, with boundary-influenced halos confined to ∼3–4 grid cells from the wrapped edges; a direct comparison of toroidal versus zero-flux Neumann versus absorbing Dirichlet BCs is positioned as future computational work for the next revision and is consistent with the proposed parameter-sweep program in Section 3.6.
6. Simulation-to-clinical timescale gap: Our model timescale (1 step ≈ 5–6 min; 500 steps ≈ 50 h; see Section 2.3) is calibrated to IFN-γ diffusion and CTL migration dynamics, not to clinical disease progression. Tumour proliferation in our model is correspondingly faster (∼one order of magnitude) than biological PCa cell-cycle durations. The simulated 15% metastatic-seeding rate over 50 h therefore cannot be directly interpreted as a clinical metastasis-onset probability, and the immunoediting dynamics observed at simulation end (500 steps) should be understood as the rapidly reached quasi-equilibrium of the immune-tumour feedback rather than the slow clinical evolution toward biochemical recurrence. Bridging from this fast (hours) timescale to the clinical (months-to-years) timescale would require either a multi-step coarse-graining scheme or a slower proliferation rate matched to PCa cell-cycle data, which we leave to future work.
7. IFN-γ concentration in dimensionless normalised units: IFN-γ concentrations in our model are reported in dimensionless normalised units rather than absolute physical concentrations (ng/mL); see Section 3.4.1 for the operational definition. While the qualitative dynamics of the model (sanctuary formation, knockout outcomes, sensitivity ordering) are independent of this choice, direct comparison of our IFN-γ field magnitudes to in vivo IFN-γ measurements requires an additional calibration step against in vitro CTL secretion-rate measurements and tumour-cell PD-L1 induction dose-response curves. Such calibration would convert our normalised units to ng/mL and permit comparison to ELISA-based IFN-γ measurements from the tumour microenvironment.
8. Spatial transcriptomics validation: Our model generates testable spatial hypotheses: PD-L1 expression should be highest at the tumour–immune interface, spatially correlated with IFN-γ signatures, with the correlation radius corresponding to the ∼30–40 μm IFN-γ diffusion length [24]. Applying the wPCF [28] to matched PCa spatial transcriptomics data would directly validate the predicted sanctuary architecture [16].
9. Necessity versus sufficiency of the static engine: Our control experiments establish that the adaptive engine is the dominant driver of tumour survival, but they also show that disabling adaptive induction ($P_{\max}=0$) does not fully revert to the static baseline (mean 1837±663 vs. static 2454.5±14.4). This suggests that either (i) the residual survival advantage in induction KO arises from other IFN-γ-mediated effects (e.g., enhanced CTL recruitment), or (ii) the static heterogeneity alone, when combined with the preserved IFN-γ field (even without PD-L1 upregulation), provides an intermediate level of protection. We have not directly tested whether static heterogeneity is strictly necessary for the adaptive engine. A confirmatory experiment in which basal *CD274* expression is set to a uniform low value (e.g., the TCGA median of 1.48 TPM) with the adaptive machinery intact would distinguish between static heterogeneity functioning as a necessary substrate for adaptive amplification versus a permissive but redundant layer. If such an arm produced survival comparable to the full adaptive model, static heterogeneity would be permissive only; if it produced near-null outcomes, static heterogeneity would be a necessary precursor. This experiment is a natural next step and would sharpen the quantitative contribution of each layer to the observed phenotype.

## 5. Conclusions

By integrating large-scale genomic data from TCGA-PRAD with a sequential multi-phase computational pipeline—from statistical analysis to discrete ABM to hybrid discrete-continuum framework—we have offered a hierarchical two-layer mechanistic reinterpretation of the cold tumour paradox in prostate cancer [1,2,19,23].

Immune evasion in PCa is not a function of bulk averages but emerges from the asymmetric interplay of two layered mechanisms. The static layer, driven by rare genomic outliers and Darwinian immunoediting, provides a reservoir of pre-adapted clones sufficient for near-complete tumour growth (mean final burden 2454.5±14.4 cells). The dominant adaptive layer, driven by the IFN-γ/PD-L1 feedback loop and phenotypic plasticity, provides a dynamic defence that creates protective sanctuaries and drives a 2.95-fold enrichment of PD-L1-high clones, generating a highly resistant population without substantially increasing absolute tumour burden. Strikingly, this adaptive engine does not require long-range diffusion of IFN-γ.

## Figures and Tables

**Table 1:** Key parameters for the discrete component of the agent-based model.

Parameter	Baseline	Range Tested	Description
grid_size	50×50	30×30–100×100	Representative 2D cross-section of TME
initial_tumour_cells	500	fixed, discrete	Density 0.20 on baseline grid (=500 cells); seed for measurable initial tumour population
initial_immune_cells	250	50–500	Density 0.10 on baseline grid (=250 cells); moderate CTL infiltration consistent with cold tumour
$P_{\mathrm{prolif}}$	0.02	fixed, discrete	Calibrated for net growth without immunity
$P_{\mathrm{seed}}$	0.001	0.0001–0.01	Lumped metastatic seeding probability
$x_{0}$	3.0 TPM	1.0–5.0	Evasion function midpoint
*k*	1.0	0.5–2.0	Evasion function steepness

**Table 2:** Key parameters for the continuum (PDE) component of the hybrid model, with provenance. Provenance categories: measured (directly from TCGA-PRAD data); literature (from published experimental measurements in comparable systems); calibrated (chosen to produce biologically plausible dynamics, then tested via sensitivity analysis).

Parameter	Symbol	Baseline (Units)	Provenance	Justification
Diffusion coefficient	*D*	0.05 grid^2^/step ≡ 5 μm^2^/step at 10 μm grid spacing	Literature	Constrained to reproduce the ∼30–40 μm IFN-γ spread measured by Centofanti et al. [24] in melanoma; value lies well below the CFL stability limit D≤0.25 [37,49].
Decay rate	δ	0.1 step^−1^ (implies half-life ≈7 steps)	Literature	Combined proteolytic degradation and cellular uptake of IFN-γ in tissue, consistent with reported cytokine half-lives in solid tumours [37,47].
Secretion rate	$S_{\mathrm{IFN}}$	10.0 arbitrary units per immune-tumour interaction per step	Calibrated	Scaled so that a single sustained CTL-tumour engagement produces local IFN-γ concentrations sufficient to trigger Hill-function induction within the characteristic ∼30–40 μm diffusion niche [24]; units are arbitrary (dimensionless within the model’s internal scale).
Maximum inducible PD-L1 expression	$P_{\max}$	15.0 TPM	Calibrated from TCGA	Chosen as an upper induction ceiling near the observed TCGA-PRAD maximum (18.50 TPM), representing the biologically plausible saturation level for IFN-γ-driven PD-L1 upregulation. Exceeded by only 1 of 554 TCGA patients (the single 18.50 TPM outlier), so represents an effective ceiling on induced expression.
Half-maximal IFN-γ concentration	*K*	5.0 units (in $S_{\mathrm{IFN}}$-scale)	Calibrated	Set to the concentration regime produced by a small cluster (2–3) of sustained CTL-tumour engagements, consistent with the switch-like induction response characterised in melanoma [21,24].
Hill coefficient	*n*	2.0 (dimensionless)	Literature	Reflects cooperativity in JAK–STAT1–IRF1 transcriptional activation of *CD274*, consistent with the switch-like response reported for interferon-driven gene induction [21,50,51].

**Table 3:** Comprehensive descriptive statistics of *CD274* (PD-L1) expression (TPM) in TCGA-PRAD primary tumour samples (n=554). The right-skewed distribution (skewness = 4.29) confirms the long-tail structure of rare high-expressing outliers that drive the static engine in the ABM.

Statistic	Value (TPM)	Notes
Central tendency and dispersion		
Sample size (*n*)	554	Primary tumour samples (sample type code 01)
Minimum	0.07	Lowest detected expression
Maximum	18.50	Single high-expressing outlier
Median	1.48	Used as cutoff in survival analysis
Mean	1.77	Higher than median, reflecting right skew
Standard deviation	1.43	
Interquartile range (Q1–Q3)	0.91–2.14	50% of cohort within ∼2-fold range
Distributional shape		
Skewness (Fisher-adjusted)	4.29	Strong right skew confirms long-tail structure
90th percentile	3.21	
95th percentile	4.10	Approximate inflection of evasion logistic ($x_{0}=3.0$)
99th percentile	7.06	Below the high-evasion threshold (9.0 TPM)
Rare-outlier counts		
Samples with TPM >6.0	9/554 (1.6%)	Above the 95th-percentile region
Samples with TPM >9.0	2/554 (0.36%)	Pre-adapted resistant reservoir, seeds static engine

**Table 4:** Univariate Cox proportional hazards model for *CD274* expression and biochemical recurrence-free survival.

Variable	HR	95% CI	*z*	*p*
CD274 (High vs. Low)	1.152	0.674–1.981	0.518	0.621

**Table 5:** Metrics of the three simulation arms at t=500 (n=50 replicates per arm).

Metric	Null Evasion	Static	Adaptive
Mean tumour count (±SD)	2455.4±12.5	2454.5±14.4	2473.3±7.8
Immunoediting ratio *	∼1.01	∼1.10	∼2.95
Evasion basis	Fixed (p=0)	Basal PD-L1 heterogeneity	IFN-γ-induced feedback
Persistence mode	None	Basal escape	Protective sanctuaries

* Ratio of median PD-L1 expression of survivors at t=500 to the initial median (1.48 TPM).

**Table 6:** Comparative analysis of *CD274* expression between initial TCGA-derived agents and survivors at t=500 for the adaptive arm.

Metric (TPM)	Initial	Adaptive Survivors	Fold Change	Biological Significance
Median	1.48	4.37	2.95	Strong enrichment of resistant clones
Mean	1.77	5.16	2.92	Selective elimination of low-evasion cells
Q3	2.14	8.52	3.98	Survivors shifted into the outlier tail

**Table 7:** Grid scale sensitivity analysis across three spatial resolutions (mean ± SD, n=20 replicates per condition).

Outcome Metric	30×30	50×50	100×100
Initial cell density (cells/area)	0.040	0.040	0.040
Final tumour burden (cells)	880.6±8.5	2445.7±14.0	9780.6±31.6
Carrying-capacity fraction (%)	97.84±0.94	97.83±0.56	97.81±0.32
Immunoediting ratio (×)	2.4±0.9	2.2±0.6	2.2±0.2
Time to equilibrium (steps)	107±6	117±6	138±17

## Data Availability

All data and simulation code are publicly available at https://github.com/ntlokwak/PCa-Immune-Evasion, accessed on 28 March 2026. The TCGA-PRAD data are accessible via the GDC Data Portal (data release v40.0), accessed on 3 August 2024.

## Supplementary Materials

The following supporting information can be downloaded at: https://www.mdpi.com/article/10.3390/biology15100806/s1, Figure S1: Proportional-hazard assumption check via Schoenfeld residuals; Figure S2: CD274 (PD-L1) BCR survival analysis: power and minimum detectable HR.

## Abbreviations

PCa	Prostate cancer
PD-L1	Programmed Death-Ligand 1
ICB	Immune checkpoint blockade
IFN-γ	Interferon-gamma
TCGA	The Cancer Genome Atlas
PRAD	Prostate Adenocarcinoma
TPM	Transcripts per million
ABM	Agent-based model
PDE	Partial differential equation
CTL	Cytotoxic T lymphocyte
TME	Tumour microenvironment
JAK	Janus kinase
STAT	Signal transducer and activator of transcription
MDSC	Myeloid-derived suppressor cell
Treg	Regulatory T cell
TAM	Tumour-associated macrophage
BCR	Biochemical recurrence
GDC	Genomic Data Commons
FTCS	Forward-Time Centered-Space
CFL	Courant–Friedrichs–Lewy
IRF1	Interferon regulatory factor 1
IHC	Immunohistochemistry
TPS	Tumour proportion score

## Appendix A

**Table A1:** Comparison of modelling approaches: this study vs. prior agent-based models of PD-1/PD-L1 interactions.

Feature	Prior ABM Approaches	This Study
PD-L1 status	Binary in Gong et al. [29]; continuous but not TCGA-calibrated in Storey & Jackson [33]; abstracted concentration in PhysiCell-based models [26]	Continuous variable sampled from empirical patient data distribution (TCGA-PRAD, n=554)
Evasion/killing rule	Fixed probabilities or rule-based parameters in Gong et al. [29] and Storey & Jackson [33]	Evasion probability dynamically calculated via logistic dose-response function of each cell’s *CD274* expression
Primary objective	General dynamics of checkpoint inhibition (Gong et al. [29]) or combination therapy response (Storey & Jackson [33])	Resolution of a specific clinical paradox (low bulk PD-L1, poor ICB response) via cellular heterogeneity and spatial dynamics
Tumour type	Generic solid tumour (Gong et al. [29]); glioblastoma (Storey & Jackson [33]); general multicellular platform (PhysiCell [26])	Explicitly parameterised for prostate cancer, a ‘cold’ tumour with a defined paradox
IFN-γ dynamics	Not explicitly modelled in Gong et al. [29] or Storey & Jackson [33]; abstracted as generic diffusible species in PhysiCell [26]	Explicit reaction-diffusion PDE with bidirectional coupling; validated via knockout controls
