# Supplementary figures and images for: Immune Evasion in Prostate Cancer: Resolving the Cold Tumour Paradox via a Hybrid Discrete–Continuum Computational Framework
Source: Biology (Basel). 2026 May 19;15(10):806. doi: 10.3390/biology15100806 (PMC13203437; doi:10.3390/biology15100806)

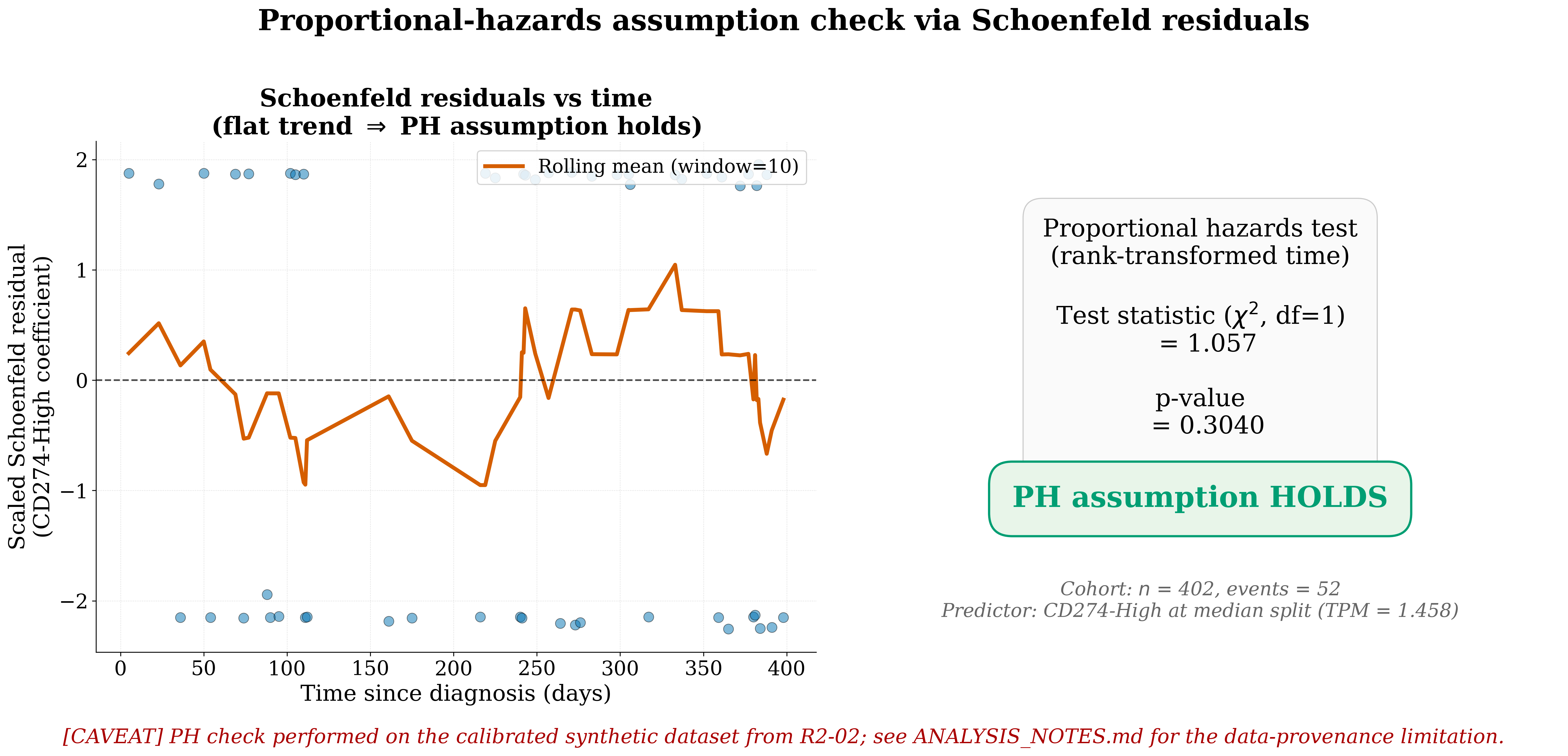

Supplement: Supplementary file 1 [file biology-15-00806-s001.zip › biology-4279220-supplementary/Figure S1 Proportional-hazard assumption check via Schoenfeld residuals.png]

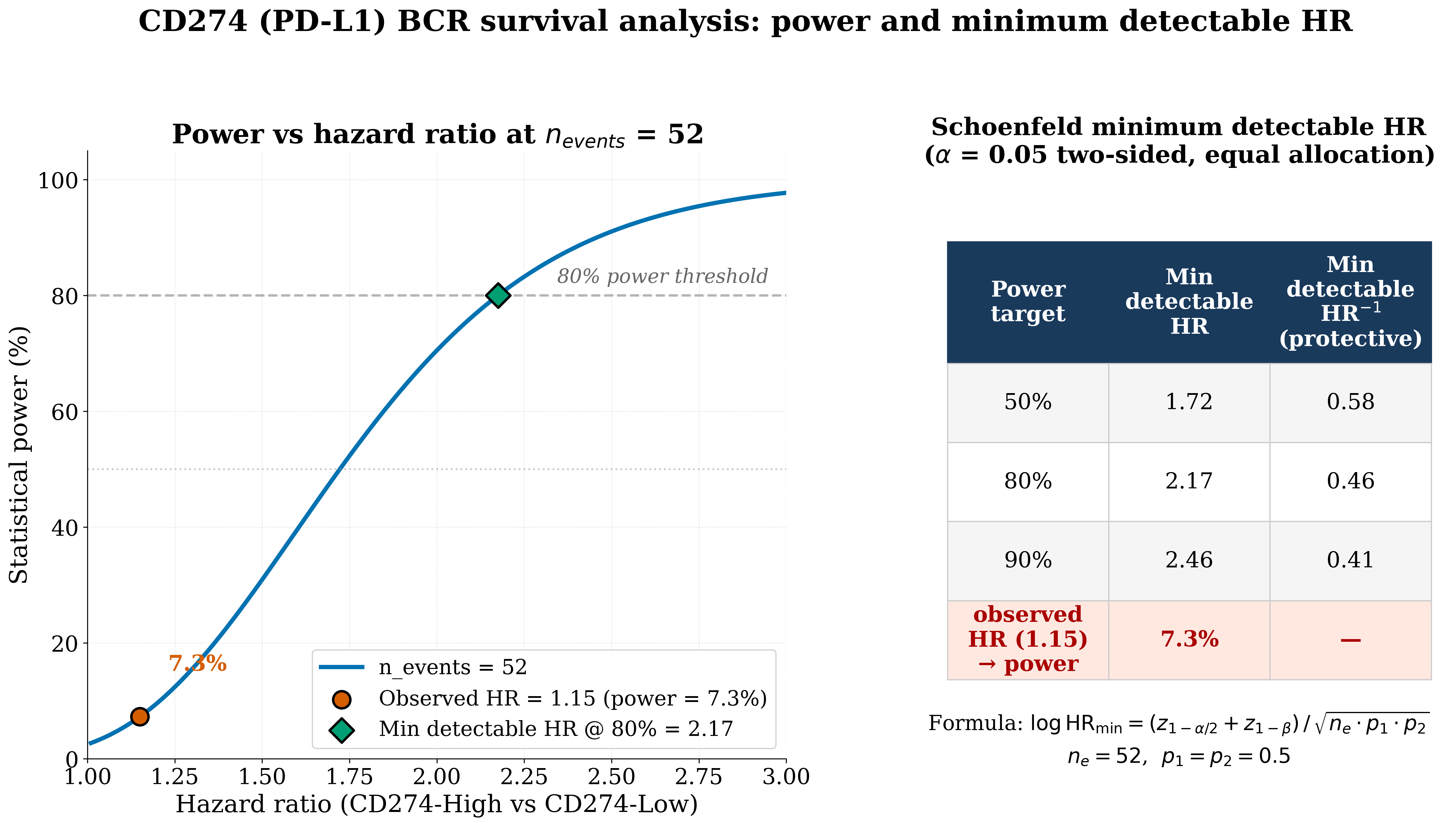

Supplement: Supplementary file 1 [file biology-15-00806-s001.zip › biology-4279220-supplementary/Figure S2 CD274 (PD-L) BCR survival analysis-power and minimum detectable HR.png]
